# New insights into Early Celtic consumption practices: Organic residue analyses of local and imported pottery from Vix-Mont Lassois

**DOI:** 10.1371/journal.pone.0218001

**Published:** 2019-06-19

**Authors:** Maxime Rageot, Angela Mötsch, Birgit Schorer, David Bardel, Alexandra Winkler, Federica Sacchetti, Bruno Chaume, Philippe Della Casa, Stephen Buckley, Sara Cafisso, Janine Fries-Knoblach, Dirk Krausse, Thomas Hoppe, Philipp Stockhammer, Cynthianne Spiteri

**Affiliations:** 1 Department of Pre- and Protohistory, University of Tübingen, Tübingen, Germany; 2 Institut für Vor- und Frühgeschichtliche Archäologie und Provinzialrömische Archäologie, Ludwig-Maximilians-Universität München, Munich, Germany; 3 Max Planck Institute for the Science of Human History, Jena, Germany; 4 Landesmuseum Württemberg, Stuttgart, Germany; 5 INRAP and ArTeHiS, UMR 6298, Villeneuve-d’Ascq and Dijon, France; 6 Institut für Archäologie Fachbereich Prähistorische Archäologie, University of Zurich, Zurich, Switzerland; 7 CCJ, UMR 7299, University of Aix-Marseille, Aix-en-Provence, France; 8 CNRS, ArTeHiS, UMR 6298, Dijon, France; 9 Landesamt für Denkmalpflege im Regierungspräsidium Stuttgart, Esslingen, Germany; Higher Institute of Applied Sciences and Technology of Gabes University of Gabes, TUNISIA

## Abstract

The rich Mediterranean imports found in Early Celtic princely sites (7^th^-5^th^ cent. BC) in Southwestern Germany, Switzerland and Eastern France have long been the focus of archaeological and public interest. Consumption practices, particularly in the context of feasting, played a major role in Early Celtic life and imported ceramic vessels have consequently been interpreted as an attempt by the elite to imitate Mediterranean wine feasting. Here we present the first scientific study carried out to elucidate the use of Mediterranean imports in Early Celtic Central Europe and their local ceramic counterparts through organic residue analyses of 99 vessels from Vix-Mont Lassois, a key Early Celtic site. In the Mediterranean imports we identified imported plant oils and grape wine, and evidence points towards appropriation of these foreign vessels. Both Greek and local wares served for drinking grape wine and other plant-based fermented beverage(s). A wide variety of animal and plant by-products (e.g. fats, oils, waxes, resin) were also identified. Using an integrative approach, we show the importance of beehive products, millet and bacteriohopanoid beverage(s) in Early Celtic drinking practices. We highlight activities related to biomaterial transformation and show intra-site and status-related differences in consumption practices and/or beverage processing.

## Introduction

Consumption practices, especially in feasting contexts, played a major role in Early Celtic life, as evidenced by a rich corpus of feasting vessels in settlements and graves [1]. Consequently, Mediterranean imports have been interpreted as an attempt by the elite to imitate Mediterranean wine feasting practices [2]. The main interpretation contends that their use indicates the adoption of a Mediterranean lifestyle north of the Alps, namely in the consumption of grape wine from Mediterranean vessels [2]. However, in recent years scholars have increasingly emphasized the transformative power of intercultural encounter and the dynamics of functions and meanings of objects in contexts of appropriation [3–5]. These approaches have also provided a basis for novel approaches to understand Celtic-Mediterranean entanglements and their impact on Early Celtic society [6–10]. We took the dynamics of cultural encounter, appropriation, practice and meaning as a starting point for understanding Early Celtic consumption practices by applying a large-scale investigation using organic residue analysis (ORA).

This study focuses on one of the key sites of the Early Iron Age (EIA) in Western Central Europe, the hillfort site of Vix-Mont Lassois (Burgundy, France). Since the start of excavations at Vix-Mont Lassois and its surroundings, hundreds of fragments of imported Mediterranean pottery have been discovered, particularly Attic black- and red-figured pottery, transport amphorae from the Greek colony of Marseille and a broad range of other Mediterranean imports–most notably the famous bronze krater from the princely burial of Vix, which is the largest metal vessel still preserved from antiquity. Moreover, the site of Vix-Mont Lassois indicates growing complexity in societies north of the Alps, especially with regard to their social and territorial structures [11]. Indeed, recent excavations have demonstrated intra-site differences in housing and social practices, which can be explained by status- and craft-related differentiations [12].

Our aim was to better understand the function and meaning of imported pottery at Vix-Mont Lassois through the application of ORA and by comparing the results to those obtained from a large corpus of locally produced wares. This comparative analysis enabled us to trace processes of appropriation far beyond previous discussions of Early Celtic culinary/dietary practices.

To date, consumption practices during the EIA in Western Central Europe are mostly discussed in terms of the techno-typology of ceramic vessels [13–15], and functions are attributed with the aid of postulated possibilities and references to ancient Greek vases in ancient literature, as well as contemporary and ethnographic analogies [16]. Few studies have targeted organic residues absorbed within ceramic assemblages during the EIA from sites located north of the Alps [17, 18].

**Fig 1:**
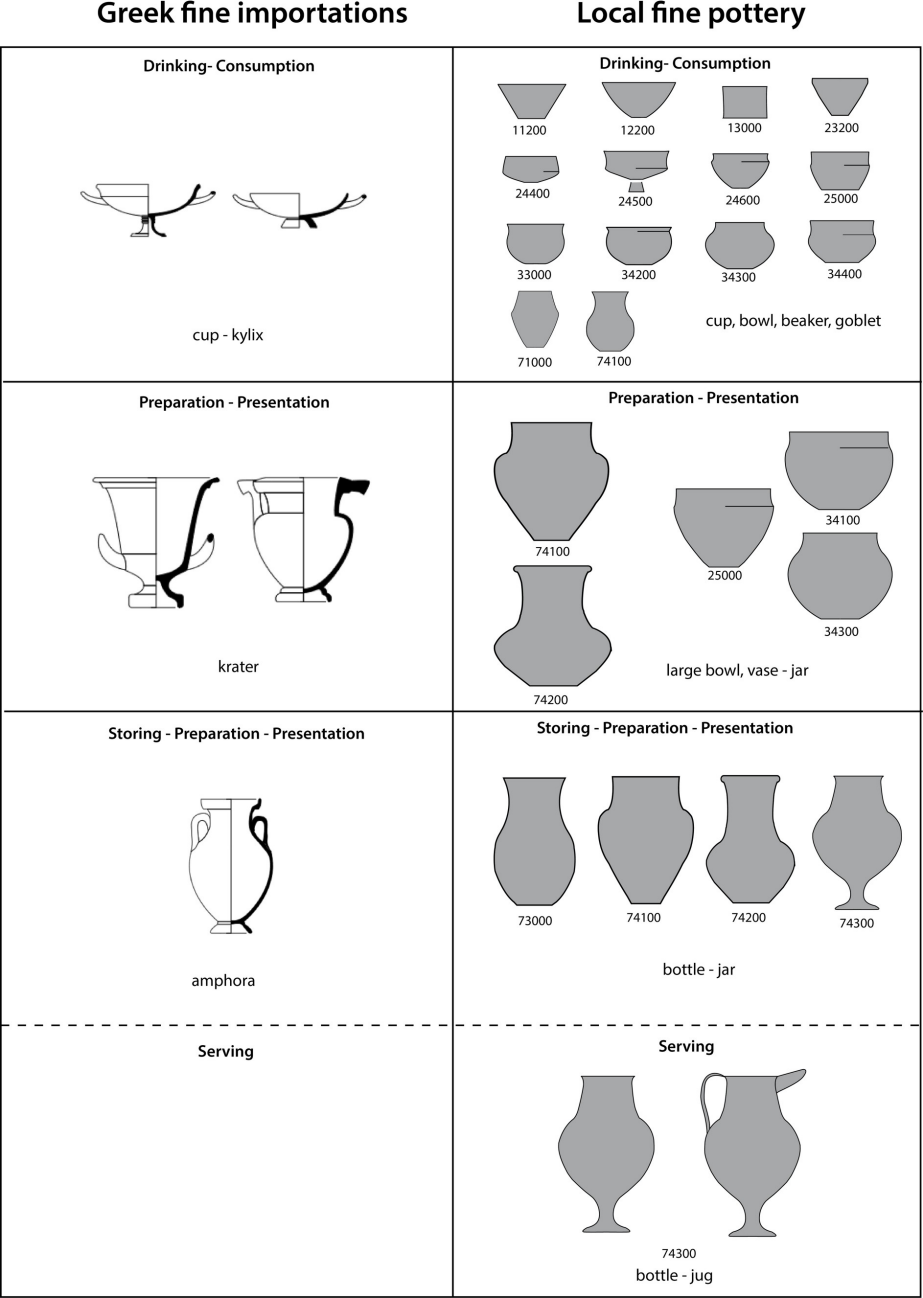
Selection of vessel forms for ORA: Imported and local vessels of similar shape and/or assumed function (courtesy of David Bardel). The numbers refer to the typology of the vessels determined by Bardel (2012).

To study consumption practices and trace processes of appropriation at Vix-Mont Lassois we investigated 99 vessels, including 16 imported Mediterranean (5 Attic cups, 5 kraters, 5 amphorae, 1 Massaliotic bowl) and 83 locally produced vessels (Fig 1, S1 Text). Locally produced fine ware vessels, selected as equivalents to the Mediterranean imports, comprised 68 handmade and wheel-turned fine wares, including 45 low forms (cup equivalents) and 23 high forms (amphorae and krater equivalents). Low forms (bowls and beakers) are associated with drinking and serving vessels, and possibly the preparation of food and drink. High forms (bottles, jars) are associated with serving, food and drink preparation, transport and storage. Lastly, 15 local coarse ware vessels were selected as a comparison to the fine wares, and in order to understand transformation/preparation processes carried out prior to consumption.

To obtain an overview of practices according to intra-site localisation and status-related uses, ceramic vessels were chosen from different contexts at Vix-Mont Lassois (S2 Text): the plateau and the lower settlements near the ramparts, both dated to Hallstatt D2-D3 (530–480 BC), and the external area, dated to Hallstatt D3-La Tène A (early 5th century BC). The architecture on the plateau was characterised by a large ostentatious apsidal building [19], and the ceramic assemblage found there was particularly rich in local fine wares and imported (Attic, Ionian, Massaliotic) vessels. Fine and coarse pottery were selected from the lower settlements near the ramparts, *Les Renards* to the east and *Champ Fossé* to the west, where domestic/craft activities have been documented [20]. Additional fine wares from the external area, *Le Breuil* (located on the plain, close to burial areas), were tested to broaden the spatial and diachronic perspective.

## Materials and methods

### Sample information

Lipids were extracted from 99 ceramic sherds kept in the Museum of the Pays Châtillonnais—Trésor de Vix (Châtillon-sur-Seine, Burgundy, France). The soil samples (controls for exogenous contamination, see S3 Text) were taken from the archaeological contexts of *Les Renards* (*fait* 211) and *Champ Fossé* (sector A) at the site of Vix-Mont Lassois (Burgundy, France). Inventory or object numbers of the Museum are provided for ceramic sherds in S1–S5 Tables.

**Fig 2:**
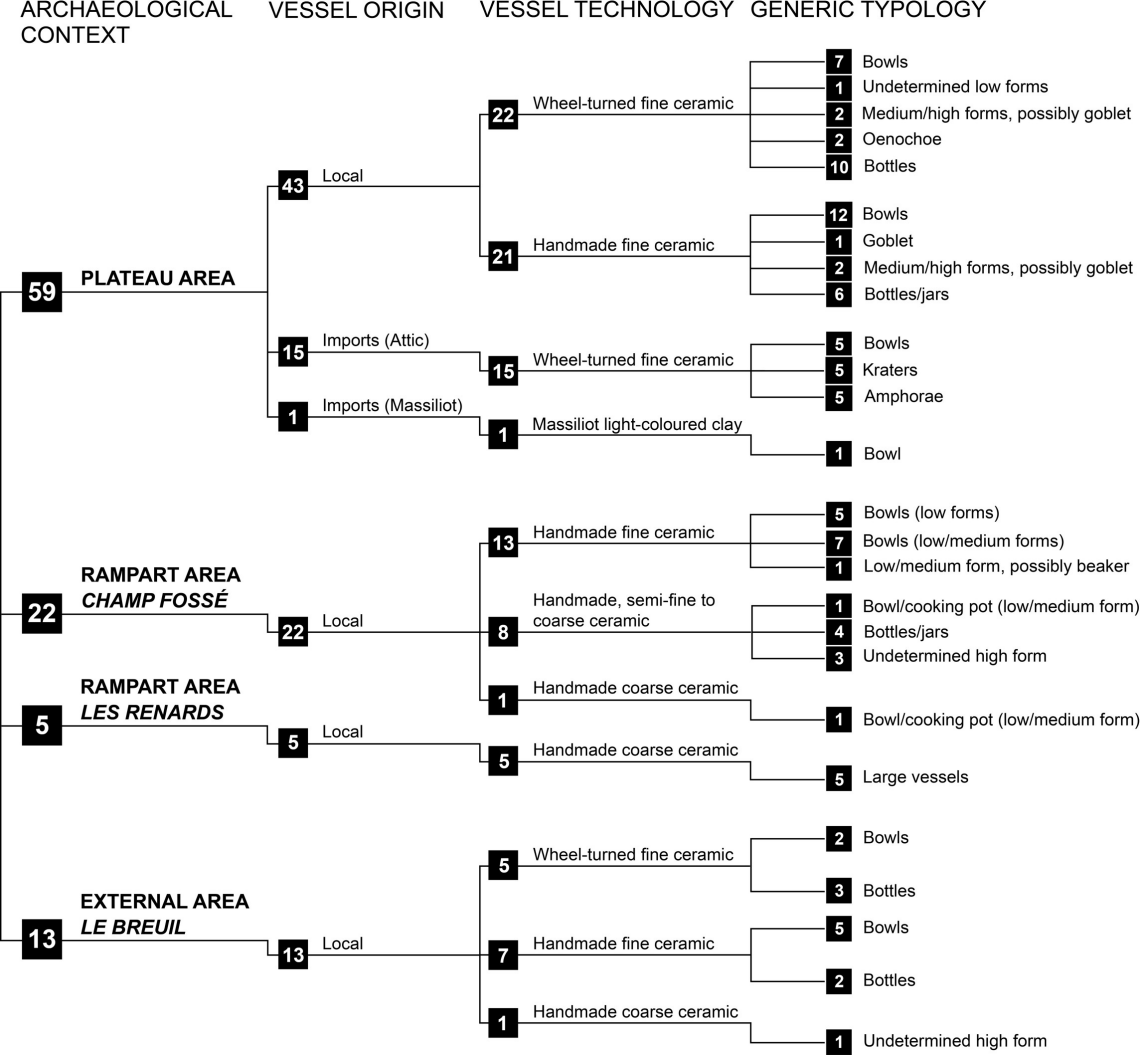
Schematic representation of the ceramics selected for ORA according to techno-typology and archaeological contexts.

The selection of archaeological samples followed a list of criteria which took into consideration the archaeological context, secure stratigraphic contexts, and the overall vessel shape (Fig 2, S1 Text, S2 Text, S1–S5 Tables). The selected vessels included imported (n = 16), local handmade (n = 41) and wheel-turned (n = 27) fine wares. 84 fine ceramic vessels, associated with drinking and serving practices by traditional typological analysis, were selected to address the question of Early Celtic drinking practices, and 15 coarse vessels associated with storage, cooking and/or transport were also selected.

### Sample treatment before GC-FID/MS and GC-C-IRMS analyses

∼2 g of potsherd were drilled following cleaning of the vessel surfaces with a modelling drill to remove any exogenous lipids (Layer 2). The lipid-rich ceramic powder collected during surface cleaning was not discarded, but retained for analysis (Layer 1). For each of the different contexts, external surfaces or soil samples taken from the burial contexts were routinely sampled to control for exogenous contamination. Lipid extraction was performed following established protocols [21, 22]. Powdered sherds were solvent-extracted (Dichloromethane-Methanol, 2:1, *v*:*v*) by ultrasonication. Aliquots of the total lipid extract were trimethylsilylated using *N*,*O*-bis(trimethylsilyl)trifluoroacetamide (BSTFA, 50 μL) and a catalytic reagent (pyridin, 4 μL) before analysis by GC and GC-MS. Aliquots of the total lipid extract were hydrolysed and methylated to obtain fatty acid methyl esters, which were then analysed by GC-FID/MS and GC-C-IRMS.

Powdered sherds were re-extracted (KOH or BF_3_ extraction) using established protocols [23, 24] to target short-chain carboxylic compounds present in high quantities in fruit products, which are insoluble in organic compounds.

Characterisation of the lipid compounds present was based on the analytical results obtained from Layer 2. Results from Layer 1 were only considered when there was no possible input from exogenous contamination, and primarily used to complete the distribution of wax esters.

### GC and GC-MS analyses

Gas Chromatography (GC) and GC-Mass Spectrometry (GC-MS) analyses were performed using an Agilent Technologies 7890B GC System series chromatograph including Agilent Technologies Capillary Flow-Technology Three-Way Splitter Kit coupled to an Agilent Technologies 5977A MSD and FID. Splitless injection was performed by a GERSTEL Multi-Purpose-Sampler and GERSTEL Cold-Injection-System 4. Samples were analysed using an Agilent J&W DB-5HT-column (15 m × 0.32 mm i.d.; 0.1 μm film thickness) and divided in two equal parts using 0.18 mm non-coated, deactivated silica capillary columns (0.66 m splitter-column to FID/ 1.52 m splitter-column to MSD) with the Three-Way Splitter Kit. The inlet temperature was ramped from 30°C to 240°C at 12°C s^-1^ (held isothermally for 5 min) and then increased to 350°C at 12°C s^-1^ (held isothermally for 10 min). The oven temperature was ramped from 50°C (held isothermally for 1 min) to 100°C at 15°C min^–1^, then to 240°C at 4°C min^–1^ and increased to 380°C at 20°C min^–1^ (held isothermally for 7 min). The analysis was carried out using helium as a carrier gas, with a split/splitless injection system, operating in the splitless mode with a purge flow of 3.0 ml min^–1^ and a constant pressure at the head of the column of 8.6667 psi. Mass spectra were acquired using electron ionization at 70 eV. The mass range was scanned from *m/z* 50–950 in 1.562 s. The temperature of the ion source was fixed at 230°C and the transfer line was set at 300°C. The temperature of the flame ionization detector (FID) was 340°C. Mass spectra were matched against authentic standards (saturated and unsaturated triglycerides, fatty acids, short-chain carboxylic compounds), published literature [25–28] and the National Institute of Standards and Technology (NIST) library, 2014 edition.

### GC-C-IRMS analyses

GC-c-IRMS measurements were carried out at the University of Liverpool. Stable carbon isotopic compositions of individual lipids were determined using a Delta V Advantage mass spectrometer (Thermo Fisher, Bremen) linked to a Trace Ultra GC with a ConFlo IV interface. Samples re-hydrated in hexane were injected in splitless mode on a J&W Scientific DB5 fused silica column (30m x 0.25mm i.d., 0.25μm film thickness). The effluent from the GC passed from the GC column immediately into and through a combustion reactor consisting of a NiO tube with CuO/NiO wires which was held at 1030°C. The effluent then passed through a water separator consisting of a Nafion tube prior to entering the MS. The GC programme was ramped from 45°C (1 minute) to 280°C at 4°C/minute and held at 280°C for 20 minutes. The injector was held at 300°C. Helium was used as the carrier gas at a constant flow of 1.4mL/min. The effluent from the GC was diverted away from the combustion reactor during the initial period of solvent elution and out of a divert valve to the atmosphere (backflush mode), while helium was passed backwards through the combustion reactor. During the solvent–divert period, CO_2_ reference gas was automatically introduced into the isotope ratio mass spectrometer in a series of pulses and its ^13^C/^12^C ratios measured. After the solvent-divert period, the effluent from the GC was allowed to enter the combustion reactor and IRMS. The IRMS automatically measured the ion intensities of *m/z* 44, 45, 46 in its three Faraday cups corresponding to ^12^C^16^O_2_, ^13^CO_2_, and ^12^C^16^O^18^O respectively. The Isodat 3 software automatically computed the ^13^C/^12^C and ^18^O/^16^O ratios of each sample peak, referenced to the standard CO_2_ gas and its known ^13^C/^12^C and ^18^O/^16^O content. Carbon isotopic compositions represent averaged values of duplicate or triplicate analyses. The CO_2_ reference gas was externally calibrated relative to Vienna Pee Dee Belemnite (VPDB) on SIRA. The results were presented in per mil (‰) relative to VPDB standard. External standards containing FAMEs (Indiana University standard F8 and FAME Standard supplied with the submitted samples) with accurately known δ^13^C values were analysed with every batch of FAME samples. The accelerating voltage was 3KV and the trap and box currents were set at 0.84mA and 0.66V respectively; the electron energy was set at 124V. The MS vacuum was 1.9 x10-6 mbar. Instrument precision was ±0.33‰.

The isotopic shift caused by the addition of a carbon atom to the fatty acids during methylation was corrected by measuring the difference between the bulk δ^13^C value obtained for the C16:0 and C18:0 fatty acid standards, and the δ^13^C measurement obtained after GC- c-IRMS analysis of the same standards. The correction factor was applied to all δ^13^C measurements of the archaeological samples analysed.

## Results and discussion

### Organic products in EIA pottery from Vix-Mont Lassois

Molecular biomarkers were preserved in most of the samples investigated (90/99). Interpretations were attempted only for samples containing ≥5μg/g of lipid (n = 85)[29, 30], with the exception of 5 samples whose overall lipid content was low but in which highly diagnostic biomarkers (miliacin; n = 2) and/or insoluble markers in the lipid fraction (short-chain carboxylic acids; n = 3) were identified in the absence of exogenous contamination (see S3 Text for control samples). Several molecular families were identified including fatty acids, *n*-alcohols, *n*-alkanes, long chain esters, triglycerides, terpenes and short-chain carboxylic compounds. These could result from over 10 natural organic products such as animal adipose fats, dairy products, beeswax, plant waxes and oils, resins, tars, grape products/wine, cereals and possibly a beverage produced from the fermentation of cereals (barley or millet) as revealed by the presence of bacteriohopanoids and supported by the archaeobotanical and use-wear records. Most of the organic substances identified were present in different combinations and within a broad range of pottery shapes and contexts. Interestingly, some of the substances identified were specific to and/or varied quantitatively according to particular vessel type and/or context (discussed below).

The presence of animal fats (n = 11), namely dairy products, ruminant adipose and porcine fats, was indicated by the distribution of saturated triglycerides (TAGs) containing up to 54 carbon atoms (n = 8), and by compound specific isotopic measurements (δ^13^C) of the palmitic and stearic fatty acids (n = 11) [25, 31](see S3 Text, S1 Table, S3–S5 Tables).

**Fig 3:**
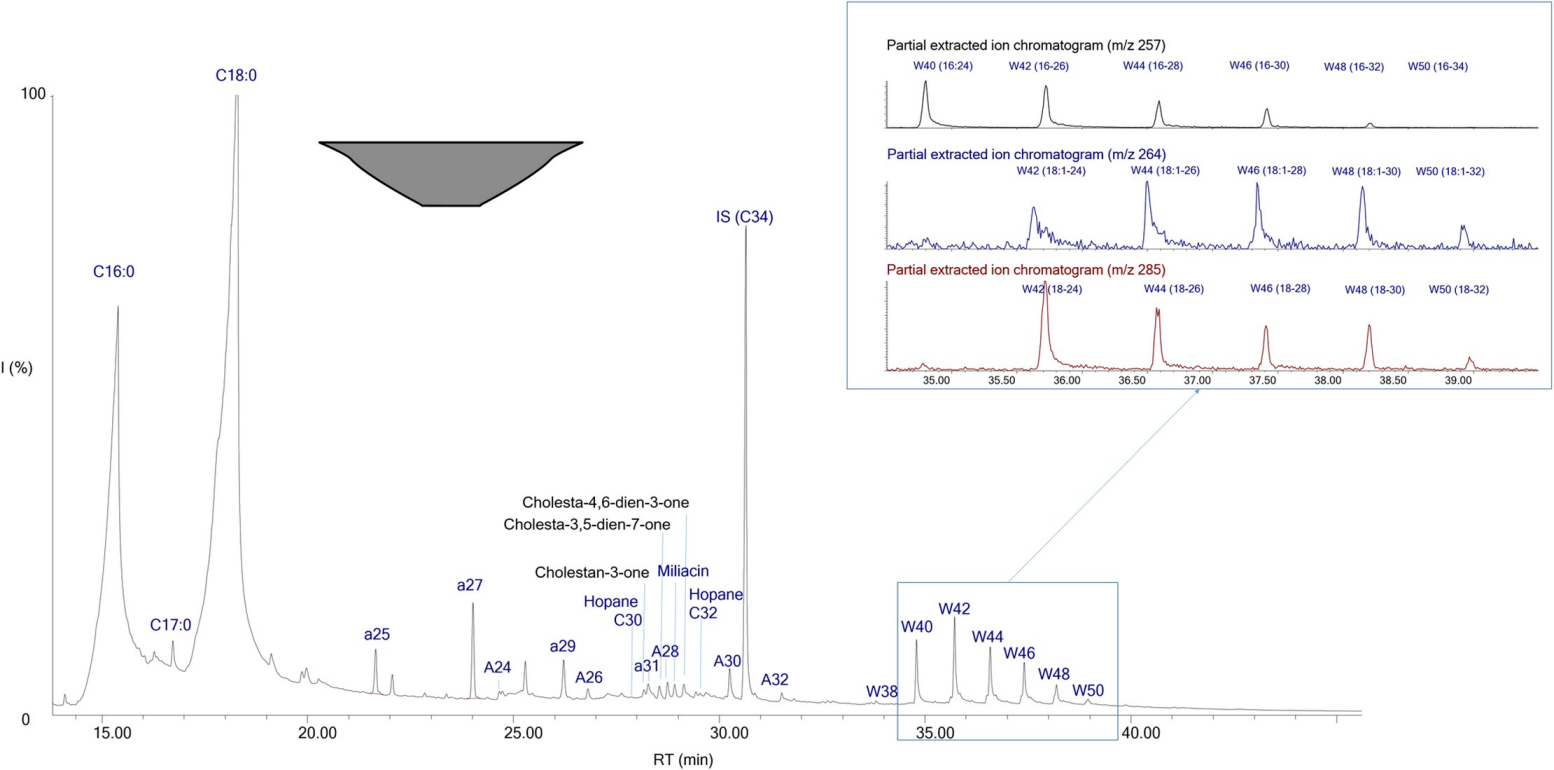
Chromatogram showing molecular constituents of a mixture containing beeswax, plant wax, millet, bacteriohopanes and animal fat (δ^13^C and Δ^13^ signatures confirming a dairy fat) in local fine bowl from *Champ Fossé* (VIX-CF-015). Cx:y = fatty acid with x carbon atoms and y representing the number of unsaturations; Ax = *n-*alcohol, ax = *n-*alkanes and Wx = long chain esters with x carbon atoms.

Long chain palmitic esters with an even number of carbon atoms ranging from C40 up to C50 were identified in a large number of vessels (n = 40) (S1 Table, S3–S5 Tables, and Fig 3). These, together with their hydrolysis products (palmitic acid and even-numbered *n*-alcohols comprising 22 to 34 carbon atoms), a series of odd-number *n*-alkanes (C_27_ major), and often with saturated long chain even-numbered fatty acids (C_24:0_ to C_28:0_) are indicative of beeswax [32, 33]. The results obtained show an extensive exploitation of beehive products, where beeswax was identified in about 50% of the local pottery tested. Beeswax is a versatile material that can be used for a variety of purposes, including diet, body care, art and technology [32–35]. Furthermore, honey, which comprises mainly fast-decaying saccharides, is unlikely to survive over archaeological timescales. Its use could however be suggested by the presence of beeswax, which is difficult to filter off completely. The high quantities of beeswax identified and the relative ease with which honey can be fermented suggests mead as a potential fermented beverages. However, no direct chemical evidence exists to support this. Hence, the presence of mead is a possibility although it cannot be conclusively determined.

**Fig 4:**
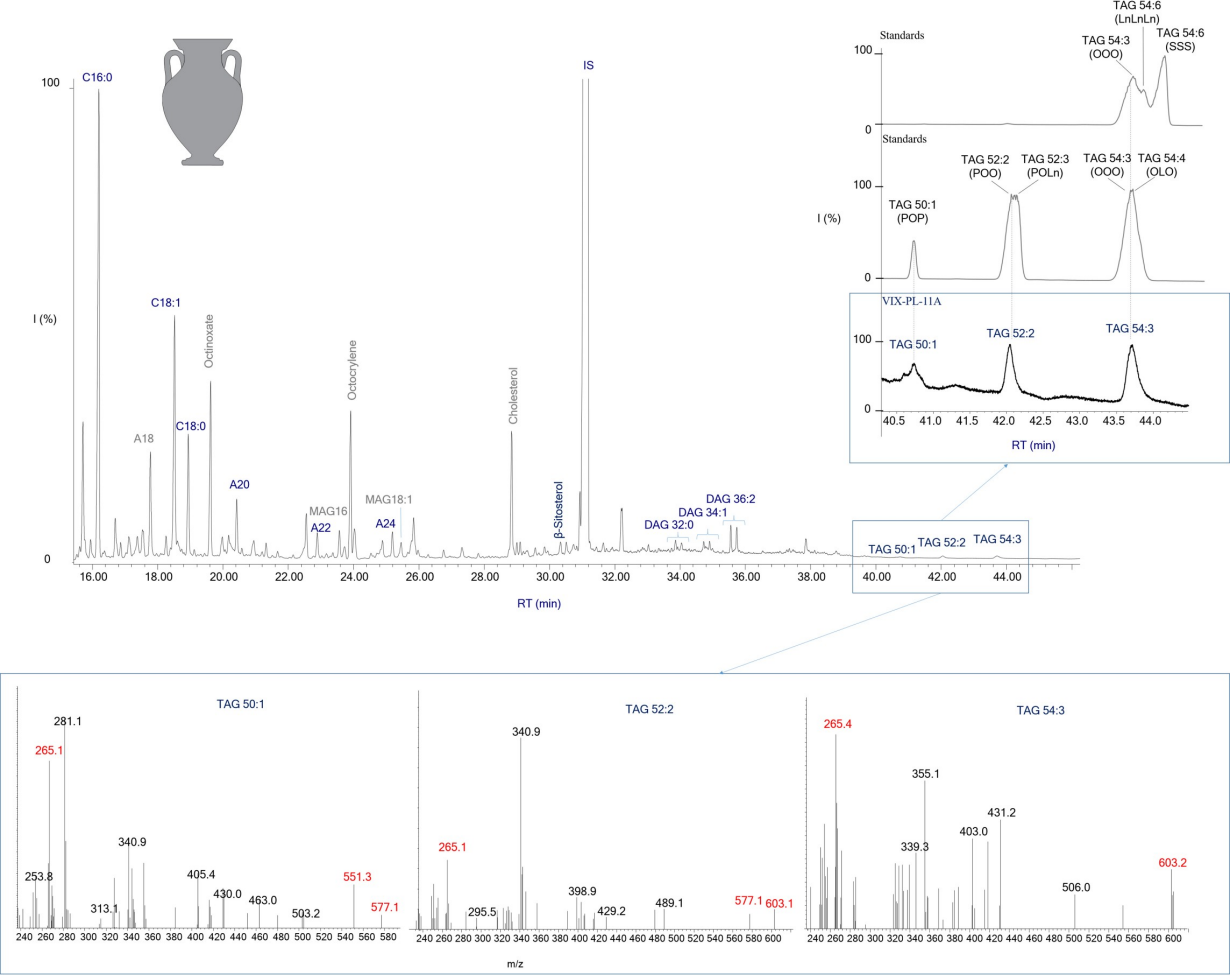
Chromatogram showing molecular markers of plant oil, type olive, in an imported Attic amphora (VIX-PL-11A) from the plateau context. Cx:y = fatty acid with x carbon atoms and y representing the number of unsaturations; Ax = *n-*alcohol with x carbon atoms; DAG = diglycerides; TAG = triglycerides. Grey: Exogenous contamination (present in the control sample).

Other long chain palmitic esters identified together with stearic and arachidic esters suggest plant waxes [36](S3 Text, S1–S5 Tables, and Fig 3) in 36 samples. These are plant by-products found in the leaves of various fruits, vegetables and cereals [36]. The botanical record attests to the possible consumption of oily plants, cereals (especially barley, millet, wheat and rye), legumes (lentil, ervil, pea, broad bean) and fruits [37, 38]. The identification of oleic acid (oleic acid ≥stearic acid; n = 20), linoleic acid (n = 12) and unsaturated TAGs (n = 3) (S3 Text) could suggest oily plants in the vessels from Vix-Mont Lassois. The high oleic to stearic acid ratios as well as the presence of unsaturated TAGs are extremely rare in archaeological samples due to decay mechanisms [29], and their identification has been associated with plant oil (31). The archaeobotanical record for local oily plants in South West Germany [37] shows the exploitation of linseed, opium poppy and camelina in Central Europe. The suite of unsaturated TAGs (54:3, 52:2 and 50:1) was identified in 3 samples (S1 Table, S3 Table and Fig 4). This TAG assemblage is present in various different plant oils. However, only a substance highly concentrated in these specific unsaturated TAGs and favourable preservation contexts will lead to their preservation and identification. Further analysis of modern reference oils was undertaken using the same analytical conditions and concentration range as in our archaeological samples. These tests showed that at least one plant oil identified at Vix-Mont Lassois does not appear to have been grown locally and is mostly consistent with a Mediterranean plant oil, particularly olive oil (Table A in S3 Text, [26]).

Miliacin, a triterpenoid marker of common millet [18, 27] (S1 Table, S3–S5 Tables, and Fig 3), was identified in more than 20% (n = 18) of the local vessels, indicating a significant consumption of this cereal. Millet can be consumed in various ways, as whole grains, porridge and cakes. However, most of these methods of consumption are unlikely to leave a chemical signal. Preparation of millet to produce porridge or a beverage, perhaps beer, is more likely to have led to the absorption of miliacin in the ceramics. The archaeobotanical record shows the presence of millet, wheat, rye and especially barley as the major cereals at Vix-Mont Lassois, and in other parts of the so-called *Westhallstattkreis*[37, 38]. The absence of wheat, barley and rye alkylresorcinols [39] from the pottery does not exclude these cereals from having been present in the vessels. Their absence could be due to the lower stability of these markers compared to miliacin, leading to an over-representation of millet.

Pinaceae resin/tar diterpenoids from the abietate family were detected in 26 vessels (S1 Table and S2 Table), and a *Pinus* origin could be specified for 6 of them due to the additional presence of pimarates and seco-abietates [28] (S3 Text). Products made from birch bark or birch sap were identified in 4 vessels through the identification of their major triterpenoid biomarkers (betulin and lupeol) [40, 41]. Additional degradation markers identified in another 3 pots show the presence of birch bark tar [42] (S3 Text).

Short-chain carboxylic acids including succinic, fumaric, malic and at times tartaric acid were identified in 20 vessels and suggest the presence of fruit products. Tartaric acid, identified in 16 vessels, is considered to be a biomarker for grape products/wine because of its higher concentration in grapes [23, 24, 43, 44] compared to other fruits available in Europe during the EIA (Table B in S3 Text, S2–S5 Tables). Grape wine consumed at Vix-Mont Lassois was probably imported from the Mediterranean area since the scant evidence of grape pips does not support the exploitation of the local wild vine. Unlike Mediterranean contexts, there is no evidence for winemaking (e.g. pressed grapes) in the Western Central European EIA [45–47].

**Fig 5:**
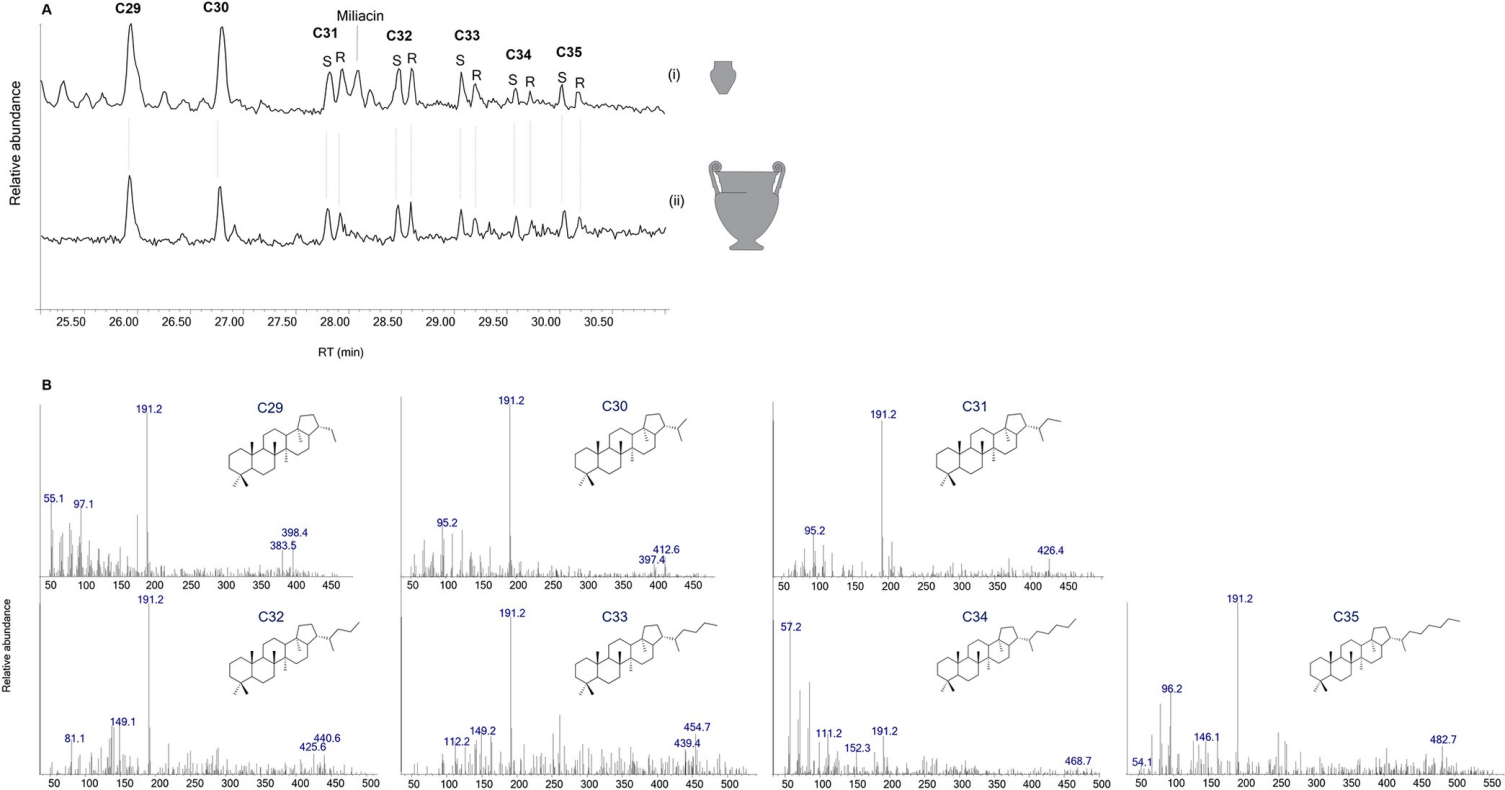
(A) Partial extracted ion chromatogram (m/z 191) showing hopane distributions (C29–C35) in (i) a local wheel-made goblet (VIX-ALT-137) and (ii) an imported Attic krater (VIX-ALT-004). (B) selected mass spectra and structural information for hopanes (C29–C35).

**Fig 6:**
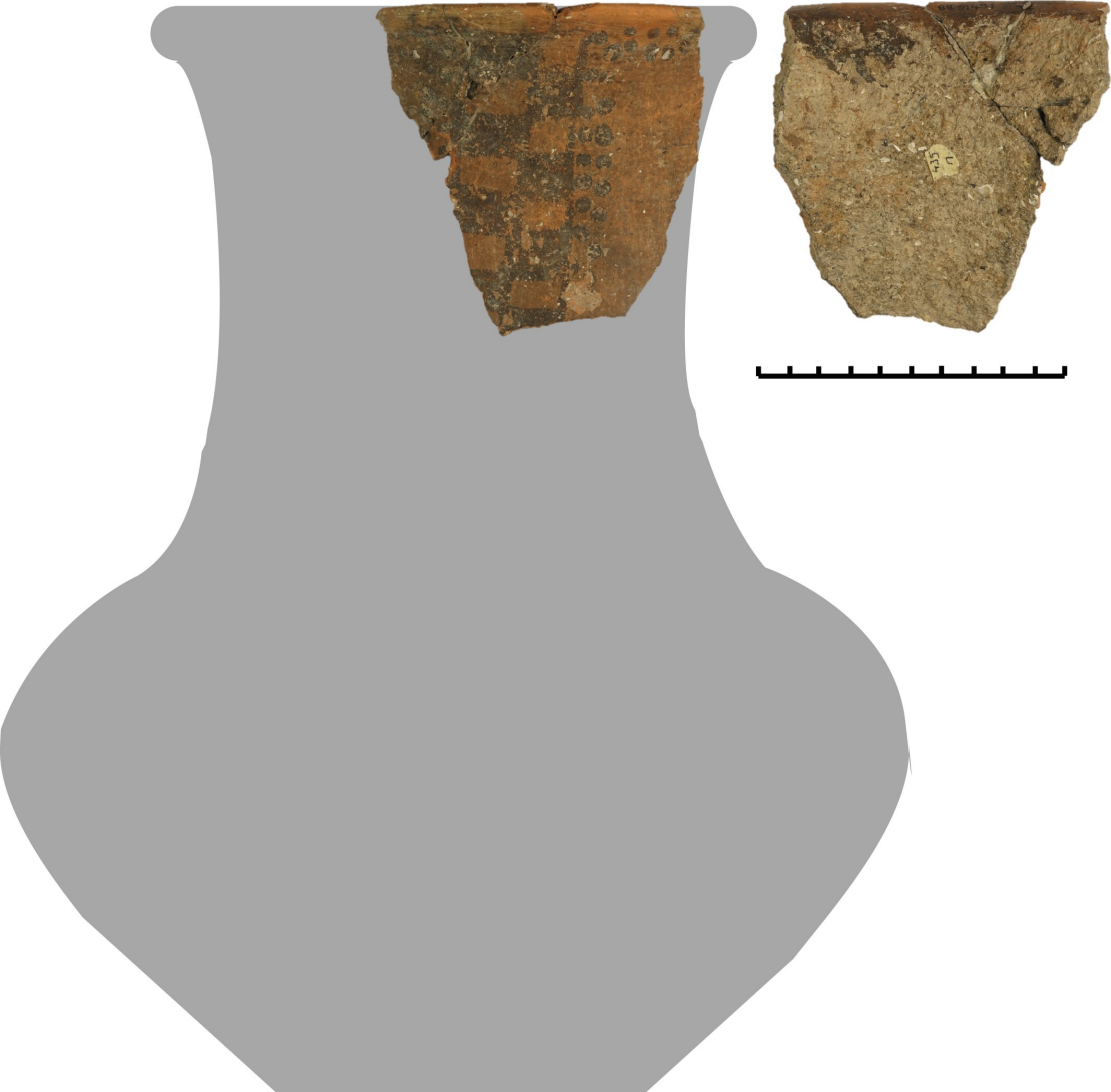
Handmade local large bottle-shaped vessel from the plateau (Vix-ALT-11) with pitting and corrosion observed on the internal wall, particularly in the upper part.

A series of compounds characterised by a base peak at m/z 191 and molecular ions (M+.) 398 (C_29_H_50_) and 412 (C_30_H_52_), and occasionally 426 (C_31_H_54_), 440 (C_32_H_56_), 454 (C_33_H_58_), 468 (C_34_H_60_) and 482 (C_35_H_62_), corresponding to hopanes, was identified in 39 vessels (S1–S3 Tables and Fig 5). These biomarkers have previously been associated with the fermentation of alcoholic beverages other than wine [48] (S3 Text), but they also occur in other substances such as bitumen [49]. Their presence at Vix-Mont Lassois was associated with a specific vessel techno-typology, and they were only identified in lipid extracts taken from the interior surface of vessels, while none were found in the corresponding exterior surfaces and sediment controls (see associated control samples in S3 Text). Our association of hopanoids with fermented beverages rather than bitumen or even plankton is supported by i) bacteriohopanoids were detected mostly in fine ceramics (absent in coarse vessels) whose shape indicates liquid consumption and which have consequently been attributed drinking and serving functions, ii) most of the bacteriohopanoid identifications were made in vessels recovered from the plateau area, which archaeological data associates with elite feasting practices and tableware and iii) use-wear analysis demonstrated pitting as a result of fermentation processes on the inner surface of experimental vessels [50], and similar evidence for pitting was observed on the neck of specific local handmade bottle-shaped vessels (Fig 6) in which bacteriohopanoids were also identified. At Vix-Mont Lassois, bacteriohopanoids were often found in association with plant oil/wax, pinaceae resin, millet and/or beeswax, showing the variety of fermented beverages which could have possibly been produced. The presence, more rarely, of animal fats and fruit/grape products may also be explained by vessel reutilisation. The more plausible interpretation is the production of beer from millet or barley, for which large quantities of botanical evidence was found on the plateau context at Vix-Mont Lassois[38].

A detailed description of the analytical results and interpretations are provided in S3 Text, and these are further contextualised in S1–S5 Tables.

### Meaning and function of imported and local vessels: Shape comparison for Hallstatt D2-D3 period (plateau and rampart contexts of *Les Renards* and *Champ Fossé*)

**Fig 7:**
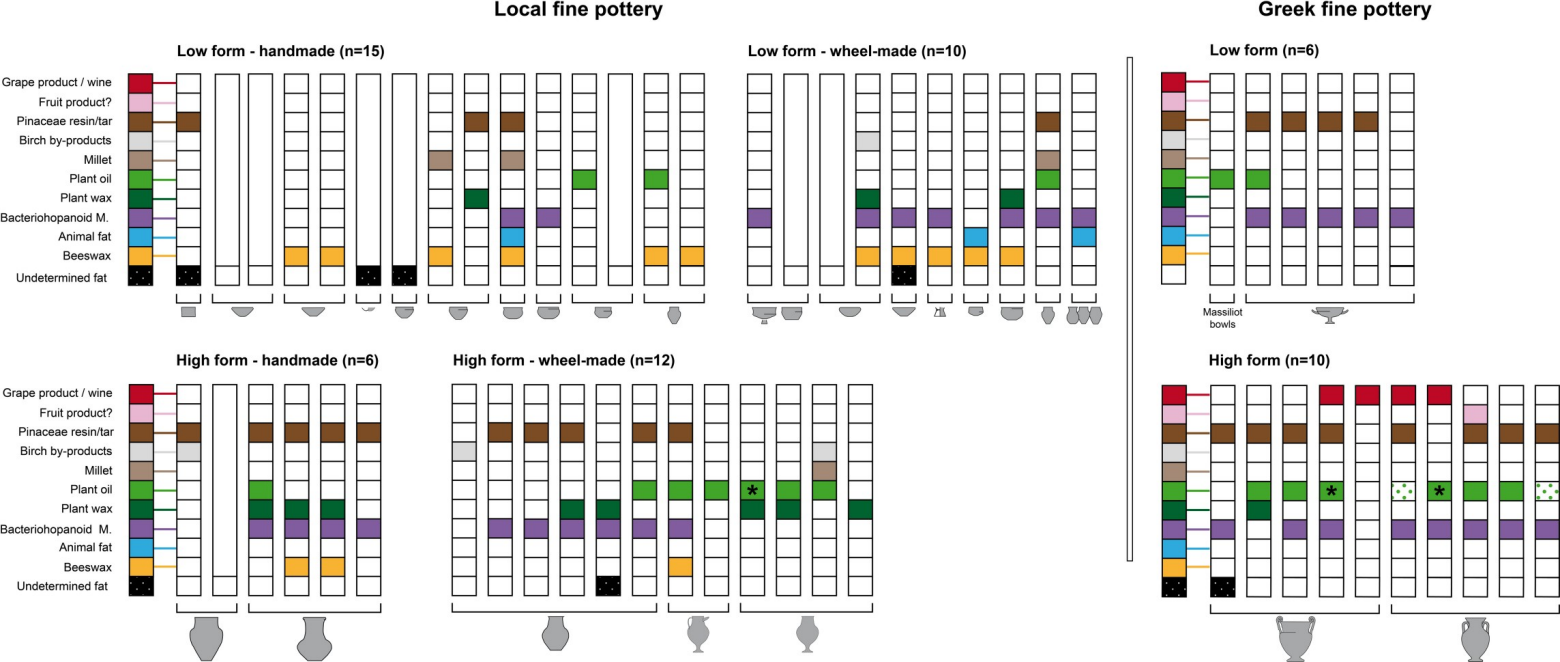
Organic substances identified in the imported and local pottery from the plateau contexts of Vix-Mont Lassois according to techno-typology. * = plant oil, type olive. Dotted green boxes = biomarkers of possible plant origin (oleic acid important, C16/C18 ≥2, absence of beeswax).

The ceramic assemblage selected from the plateau context comprises most of the fine ware shapes tested and allows for a comparative study of imported and locally produced wheel-made and handmade vessels (Fig 7). Attic sherds are only present in this context. Results for the rampart areas allow a contemporaneous comparison.

#### Fine wares: High forms

The high forms of the selected fine wares include 5 Attic amphorae, 5 Attic kraters and 18 locally produced jar/bottle-shaped vessels. They are associated with a liquid or semi-liquid content. Beeswax is significantly rare in local and absent in Mediterranean vessels (present in 3/18 locally produced bottles on the plateau). Conversely, bacteriohopanoid markers were identified in all 5 Attic amphorae tested and in 3 of the 5 Attic kraters. These markers were also present in 10 of the 18 locally produced bottles from the plateau, often together with plant wax and/or oil. Pinaceae resin/tar was identified in imported (n = 8) and local high forms (n = 10), *quasi* systematically together with bacteriohopanoid markers (7/8 and 9/10) but rarely with wine. Plant oil (including a Mediterranean plant oil, type olive) is the other main substance identified in 3 of the Attic amphorae, 3 of the Attic kraters, and in local wheel-made bottle-shaped wares (n = 6/12). This confirms the postulated function of Attic and local wheel-made high forms as storage/serving vessels for different types of liquids. Furthermore, grape wine (probably Mediterranean) was identified in 2 of the Attic amphorae and 2 of the Attic kraters, but absent in the local fine high forms from the plateau. In contrast, a birch by-product, possibly sap or “juice”, is only observed in local vessels as a unique substance (n = 2) and once together with millet, bacteriohopanoid markers, plant oil and wax. Frequent pitting and corrosion were observed on the internal walls of some of the local large handmade bottle-shaped vessels, particularly in the upper part (n = 4, Fig 6), together with bacteriohopanoid markers. Due to the use of specific corrosive agents, pitting occurs inside these bottles/jars, which are thought to have been used for the preparation of liquid/semi-liquid products (S1 Text). Two hypotheses can be put forward, i) the preparation and storage of cured products, and ii) the preparation of fermented beverages, especially beer [50]. The presence of bacteriohopanoid markers in these bottles with corroded internal walls supports the hypothesis of beer preparation, possibly including additives as suggested by the presence of beeswax (n = 2/4), plant wax (n = 3/4) and Pinaceae resin (4/4). Plant wax could have pertained to the original fermented plant-product. Although no specific cereal molecular markers were identified, the large quantities of barley seeds recovered from the same contexts [38] supports barley beer production.

#### Fine ware: Low forms

Comparative analyses of the fine low forms (n = 44), which are generally assumed to have been drinking vessels and/or food preparation-presentation vessels (particularly the larger bowls), enable a deeper understanding of drinking practices from the plateau and rampart areas in relation to their origin (local or Mediterranean) and shape. Most notable is the *quasi* absence of wine signatures in the low forms of the Attic and local fine wares from the plateau and rampart areas; only 2 bowls–both local and from the rampart area–tested positively for wine. Theoretically, this could be explained by degradation and/or a short use-life of the pottery leading to lower absorption, which is not conducive to preservation over long time-scales. In addition to the challenges surrounding preservation and multiple reuses of these low forms, it is possible that perishable or recycled metal containers may also have been used to drink wine. However, the 5 Attic bowls from the plateau contained bacteriohopanoid products, 4 of which also contained Pinaceae resin. This indicates a specific function dedicated to the consumption of possibly a cereal-based fermented beverage(s). Similarly to the Attic bowls, local wheel-made bowls and bottle-shaped miniatures are frequently associated with the consumption of alcoholic beverages (n = 7/10), whereas these drinks are present in less than 50% of the handmade low forms (plateau and *Champ Fossé*). On the plateau, plant oil was identified in 1 of the Attic bowls, in 2 local bottle-shaped miniatures as well as in the Massaliotic bowl and in 1 local bowl as a single substance. Locally produced fine low forms contained additional products, namely beeswax, millet, plant wax, animal fats and wine (2 bowls from *Champ Fossé*). Among the largest handmade bowls from *Champ Fossé* (8 low/medium forms), which were probably used for the consumption of liquids or as small storage and/or preparation-display vessels, beeswax (n = 6), millet (n = 4) and plant wax (n = 4) were the main substances identified. Bacteriohopanoid markers were also found (n = 3), as were animal fats (1 dairy and 2 adipose fats), the highest proportion of which were identified in this fine ware category. Animal fats were always associated with beeswax and millet, and often with bacteriohopanoid markers and plant wax, suggesting multi-ingredient dishes and/or functional polyvalence of vessels. The presence of porcine and ruminant adipose fats in 2 bowls could suggest the consumption of meat, sauce or soup.

#### Coarse wares

Local coarse and semi-fine to coarse wares, associated with food preparation, cooking, storage or transport (n = 15), were characterised by the diversity of their content due to potential mixtures of different products and/or reuse. Bacteriohopanoid beverages were absent (except in one semi-fine to coarse ware) while beeswax, animal fats, plant wax and millet were consistently identified, which suggests that they were important food products. The 5 large jars, associated with storage and/or food preparation, recovered from the rampart area of *Les Renards* presented unexpected mixtures, including plant waxes (n = 4), dairy fats (n = 3), beehive products (n = 4), millet (n = 2), imported wine (n = 3) and possibly other fruit-based fermented beverages (n = 2), with the wine markers being remarkably significant. Four large high form vessels from *Champ Fossé* could potentially have been used for storing a millet by-product since miliacin was present as a single substance in 2 vessels, while another 2 vessels contained chemical signatures for millet, plant oil and beeswax.

### Spaces of consumption

#### Plateau contexts

The fine wares studied from the elite-related plateau revealed specific consumption practices including the highest proportion of bacteriohopanoid alcoholic beverage(s) (44% local and 81% imported wares), plant oil (23% local and 50% imported vessels), a birch-derived beverage, and Pinaceae resin. These results, as well as the presence of wine in the Attic amphorae and kraters, further corroborate the proposed function of the tested imported pottery from this context as drinking vessels containing primarily alcoholic drinks. This points to the importance of the various alcoholic beverages for EIA individuals living on the plateau–most of them probably with high status positions [51]. This is supported by the high quality of the vessels, which further points to social specialisation.

#### Lower settlements near the ramparts

The ceramic assemblage from the domestic/craft-related rampart areas was characterised by the highest presence of animal products (>30% animal fats and >60% beeswax), plant waxes (59%) and millet (48%). Most surprising is the presence of wine in local vessels, as this is so far the only evidence for wine in locally produced pottery at Vix-Mont Lassois during Hallstatt D2-D3. Given the context and vessel techno-typology, imported wine was probably part of storage and/or culinary practices at *Les Renards*. The location of *Les Renards* is significant because it lies close to the Seine River and the communication route on the way to the plateau, making it a perfect place for storing and processing imported food and drink, while the absence of bacteriohopanoid markers from this area seems to indicate that there was no production of such fermented beverages.

**Fig 8:**
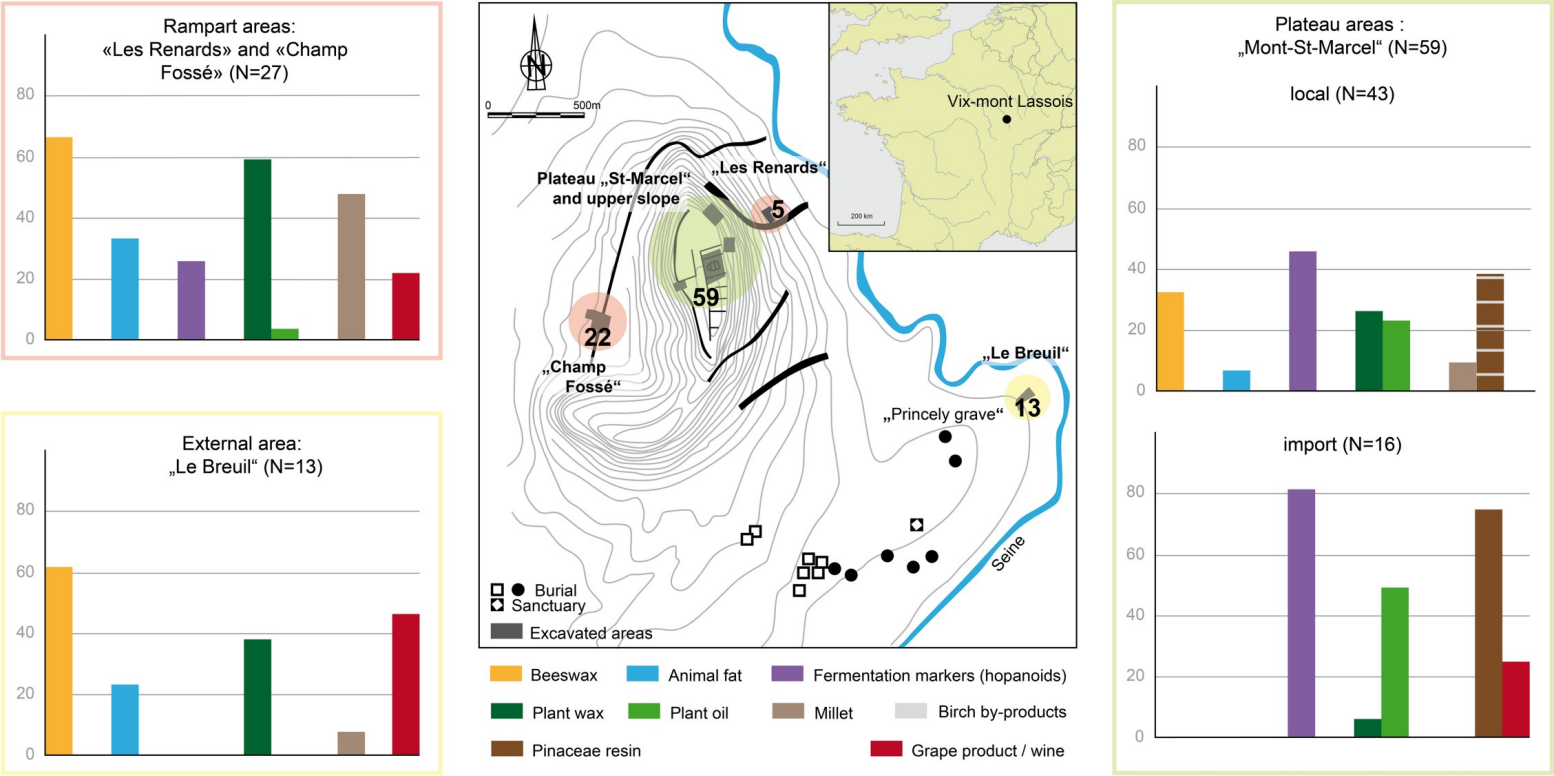
Organic substances identified in the pottery from Vix-Mont Lassois according to the different contexts from which they were recovered.

A similar range of products was identified in the fine and coarse wares at *Champ Fossé* (S3 Table). Consumption practices seem to be linked to space (Fig 8) rather than the techno-typology of the vessels–except for the consumption of bacteriohopanoid beverage(s), which are prevalent in fine ceramics. The context of *Champ Fossé* is characterised by the consumption and/or production of millet by-products (millet identified in ≥30% of all shapes and 67% of the fine handmade bowls), particularly from the first phases of occupation, namely before the construction of the rampart when 70% of the pottery contained millet signatures. The presence of miliacin in 2 large coarse pots as a single content could indicate storage of millet by-product(s). When present in fine handmade bowls, millet was always found together with beeswax (n = 8), and in 4 of these 8 vessels bacteriohopanoid markers were also present, suggesting millet beer.

#### Context of *Le Breuil*, an external settlement dating to Hallstatt D3-La Tène A

The fine wares recovered from the external settlement of *Le Breuil* are typologically similar to the locally produced wheel-turned and handmade vessels from the plateau. However, their organic contents vary significantly. Of interest is the presence of wine in 4 of 5 fine handmade bowls and in 1 wheel-made bottle. The presence of ruminant adipose fat in 1 of the bottles is also surprising as it is an unusual content for this vessel type. In contrast, bacteriohopanoid beverages and plant oil are entirely absent from *Le Breuil*. We identified beeswax in more than 60% of the different vessel types tested, and it appears more frequently in the high forms (n = 3 of 5), similarly to other contexts at Vix-Mont Lassois. Several hypotheses can be brought forward to explain the specific patterns of consumption at *Le Breuil*: i) the slightly later date of this area could indicate a change in consumption practices and/or ii) the results derive from the specific status of the area which is located close to the necropolis.

## Conclusion

### Specialisation of activities related to drinking practices at Vix?

The identification of fermented beverages at Vix-Mont Lassois underlines the importance of alcoholic beverages in Early Celtic society. The results shed new light on Early Celtic drinking practices and the production of these beverages, as suggested by ORA and corrosion on the inside of the neck of large bottles. Furthermore, we identified status- and/or space-related practices of consumption and/or preparation of fermented beverages. In these contexts, alcoholic beverages had the potential to shape, enforce and transform identities within the society [52]. The production and consumption of a specific beer (possibly from barley but not from millet) and birch-derived beverages seem to be related to individuals (probably having high status positions) living on the plateau and/or the individuals who had access to feasting events taking place there. Drinking on the plateau most probably also included the consumption of grape wine from Mediterranean feasting vessels, which would indicate the appropriation of Mediterranean feasting practices to some extent and its limitation to a specific group of people within this society. There is very little direct evidence that grape wine was consumed from drinking vessels, except for the two locally made bowls from *Champ Fossé*. Furthermore, the Mediterranean feasting vessels on the plateau were also used for the consumption of beer possibly made from barley and not limited to grape wine. In contrast to (possibly) barley beer, millet beer was rarely consumed on the plateau. Its consumption appears to be associated with individuals likely of lower status who lived and/or worked in the domestic/craft-related rampart areas, especially at *Champ Fossé* and *Les Renards*. It is difficult to identify the potential consumers of the wine identified by ORA in the large cooking and/or storage vessels at *Les Renards*. It could have been consumed by the craftspeople in this area or it could also have been stored here for later conspicuous consumption in other areas of the site e.g. by the elite on the plateau. The possibility that wine was being consumed by craftspeople at *Les Renards* and *Champ Fossé* is of particular relevance. If we hypothesise that the consumption of wine in the Celtic society at Vix-Mont Lassois was accessible to all, and not restricted to Attic imports in the elite context of the plateau, it is the act and place of consumption, not wine as a substance, that might have influenced the formation and identification of social groups, and their status within local society.

The high quantities of beeswax identified in the pottery highlights the importance of this resource and suggests a strong local management of beehives and maybe even bee domestication. Even if the function of the beeswax identified in the local vessels is still difficult to interpret, there is no doubt that beehive products played a crucial role in drinking practices–irrespective of whether it was consumed as flavouring agent, sweetener or whether it was used as a sealant to waterproof vessels as has previously been identified in Mediterranean protohistoric contexts [34]. The use of beehive products is remarkably evident in the fine low forms, especially the wheel-made bowls in which beeswax was almost always detected. The absence of beeswax in imported drinking and serving vessels confirms its local specificity.

### Appropriation of Mediterranean products, vessels and practices

The identification of plant oil (type olive) and wine markers in both Attic and local wares (19/99 vessels) demonstrates the importation of organic products of Mediterranean origin and their appropriation in Early Celtic consumption practices. Although Pinaceae are abundant in Central Europe, pine resin was thus far only known in Mediterranean consumption practices (for flavouring and/or as antibacterial agent). The site of Vix-Mont Lassois provides evidence of this practice north of the Alps with Pinaceae resin/tar identified in 12 of 15 Attic wares as well as 14 of the 43 local fine wares from the plateau context, and mostly occurring together with fermentation markers. Similar functions were observed in local wheel-made and Mediterranean high shapes, i.e. the consumption of fermented beverages and plant-derived oils. We could also identify a difference in the use of similar shapes depending on their origin: on the plateau, wine was only identified in Attic vessels whereas beeswax, birch-derived products and millet only in the local fine wares. Thus, we find an interesting continuity in the use of some of the vessel types associated with wine consumption in both the Mediterranean and the Northern Alpine region (kraters, amphorae), while the Mediterranean wine drinking vessels (cups, kylikes) vessels acquired a new function demonstrating dynamics of intercultural encounter.

## Supporting information

S1 Text Selected vessels and postulated functions according to techno-typology. (DOCX)

S2 Text The investigated contexts of Vix-Mont Lassois. (DOCX)

S3 Text Results and interpretation of substances: Detailed discussion. (DOCX)

S1 Table Local vessels from the plateau context. List of samples including the analytical results and their interpretation arranged according to context.
(XLSX)

S2 Table Imported vessels from the plateau context. List of samples including the analytical results and their interpretation arranged according to context.
(XLSX)

S3 Table Local vessels from Champ Fossé. List of samples including the analytical results and their interpretation arranged according to context.
(XLSX)

S4 Table Local vessels from Les Renards. List of samples including the analytical results and their interpretation arranged according to context.
(XLSX)

S5 Table Local vessels from Le Breuil. List of samples including the analytical results and their interpretation arranged according to context.
(XLSX)

## References

[1] Krausse D. Hochdorf III. Das Trink- und Speiseservice aus dem späthallstattzeitlichen Fürstengrab von Eberdingen-Hochdorf (Kr. Ludwigsburg). Forschungen und Berichte zur Vor- und Frühgeschichte in Baden-Württemberg 64 Stuttgart: Konrad Theiss; 1996.

[2] Kimmig W. Die griechische Kolonisation im westlichen Mittelmeer und ihre Wirkung auf die Landschaften des westlichen Mitteleuropa. Jahrbuch Römisch-Germanisches Zentralmuseum. 1983;30:5–78.

[3] Thomas N. Entangled Objects: Exchange, Material Culture and Colonialism in the Pacific Cambridge u. London; 1991.

[4] Stockhammer PW. Performing the Practice Turn in Archaeology. Transcultural Studies. 2012;1:7–42.

[5] Maran, J., Stockhammer PW. Materiality and Social Practice. Transformative Capacities of Intercultural Encounters. Papers of the Conference, Heidelberg, 25th–27th March 2010. Oxford: Oxbow; 2012.

[6] Stockhammer PW, Athanassov B. The Westhallstattkreis as Spaces of Contact. Tempo. 2018; 24(3):621–33.

[7] Dietler M. Archaeologies of Colonialism: Consumption, Entanglement, and Violence in Ancient Mediterranean France. Berkeley: University of California Press; 2010.

[8] Guggisberg M. Die Hydria von Grächwil. Zur Funktion und Rezeption mediterraner Importe in Mitteleuropa: Verlag Bernisches Historisches Museum; 2004.

[9] Kistler E. Großkönigliches „symbolon”im Osten–exotisches Luxusgut im Westen. Zur Objektbiographie der achämenidischen Glasschale aus Ihringen. In: Lang M, Gufler B, editors. Die vielfältigen Ebenen des Kontakts: Interkulturelle Begegnungen in der Alten Welt: Wiesbaden; 2010 p. 63–95.

[10] Verger S. Partager la viande, distribuer l’hydromel. Consommation collective et pratique du pouvoir dans la tombe de Hochdorf In: Krausz S, Colin A, Gruel K, Ralston I, Dechezleprêtre T, editors. Mélanges en l’honneur d’Olivier Buchsenschutz. Bordeaux: Ausonius Éditions; 2013 p. 495–504.

[11] Brun P, Chaume B. Une éphémère tentative d’urbanisation en Europe centre-occidentale durant les VIe et Ve siècles av. J.‑C.? Bulletin Société Préhistorique Française. 2013; 110(2):319–49.

[12] Chaume B. Le complexe aristocratique de Vix/le mont Lassois. Bull Arch et Hist Châtillonnais 2013;5(31–45).

[13] Balzer I, editor Die Drehscheibenkeramik aus den Altgrabungen des Mont Lassois–ein Zwischenbericht La céramique hallstattienne de France orientale: approches typologique et chrono-culturelle Actes du colloque international de Dijon; 2009; Dijon: Editions universitaires.

[14] Bardel D. Société, économie et territoires à l’âge du Fer dans le Centre-Est de la France Analyse des corpus céramiques des habitats du Hallstatt D–La Tène A (VIIe—Ve siècle av. J.-C.): Université de Bourgogne; 2012.

[15] Augier L, editor La céramique façonnée au tour: témoin privilégiée de la diffusion des techniques au Hallstatt D2-D3 et à la Tène A-B1 L’âge du Fer en Aquitaine et sur ses marges Mobilité des hommes, diffusion des idées, circulation des biens dans l’espace européen à l’âge du Fer Actes du 35e Colloque international de l’AFEAF; 2013; Pessac: Féd. Aquitania.

[16] Richter GMA, Milne MJ. Shapes and names of Athenian vases. New York: New York: The Metropolitan Museum of Art; 1935.

[17] Copley MS, Berstan R, Dudd SN, Straker V, Payne S, Evershed RP. Dairying in antiquity. I. Evidence from absorbed lipid residues dating to the British Iron Age. Journal of Archaeological Science. 2005;32(4):485–503.

[18] Heron C, Shoda S, Breu Barcons A, Czebreszuk J, Eley Y, Gorton M, et al First molecular and isotopic evidence of millet processing in prehistoric pottery vessels. Scientific Reports. 2016;6:38767 10.1038/srep38767 28004742 PMC5177950

[19] Chaume B, Mordant C. Le complex aristocratique de Vix In: Chaume B, Mordant C, editors. Nouvelles recherches sur l’habitat et le système de fortifications. Dijon: Editions universitaires; 2011 p. 867.

[20] Winkler A, Della Casa P. Une zone artisanale hallstattienne sur le site princier de Vix (Côte-d’Or) au lieu-dit Les Renards. Bilan intermédiaire In: Marion S, Deffressigne S, Kaurin J, Bataille G, editors. Production et proto-industrialisation aux Âges du Fer Perspectives sociales et environnementales Actes du 39e colloque international de l’AFEAF; 4–17 mai 2015; Nancy. Bordeaux: Ausonius éditions; 2017 p. 693–700.

[21] Mottram HR, Dudd SN, Lawrence GJ, Stott AW, Evershed RP. New chromatographic, mass spectrometric and stable isotope approaches to the classification of degraded animal fats preserved in archaeological pottery. Journal of Chromatography A. 1999;833(2):209–21.

[22] Debono-Spiteri C, Gillis RE, Roffet-Salque M, Navarro LC, Guilaine J, Manen C, et al Regional asynchronicity in dairy production and processing in early farming communities of the northern Mediterranean. Proceedings of the National Academy of Sciences of the United States of America. 2016; 113(48):13594–9. 10.1073/pnas.1607810113 27849595 PMC5137723

[23] Pecci A, Giorgi G, Salvini L, Cau Ontiveros MÁ. Identifying wine markers in ceramics and plasters using gas chromatography–mass spectrometry. Experimental and archaeological materials. Journal of Archaeological Science. 2013;40(1):109–15.

[24] Garnier N, Valamoti SM. Prehistoric wine-making at Dikili Tash (Northern Greece): Integrating residue analysis and archaeobotany. Journal of Archaeological Science. 2016;74:195–206.

[25] Regert M. Analytical strategies for discriminating archeological fatty substances from animal origin. Mass Spectrometry Reviews. 2011;30(2):177–220. 10.1002/mas.20271 21337597

[26] Garnier N, Rolando C, Høtje JM, Tokarski C. Analysis of archaeological triacylglycerols by high resolution nanoESI, FT-ICR MS and IRMPD MS/MS: Application to 5th century BC–4th century AD oil lamps from Olbia (Ukraine). International Journal of Mass Spectrometry. 2009;284(1–3):47–56.

[27] Jacob J, Disnar J-R, Arnaud F, Chapron E, Debret M, Lallier-Vergès E, et al Millet cultivation history in the French Alps as evidenced by a sedimentary molecule. Journal of Archaeological Science. 2008;35(3):814–20.

[28] Helwig K, Monahan V, Poulin J. The identification of hafting adhesive on a slotted antler point from a southwest Yukon ice patch. American Antiquity. 2008;73:279–88.

[29] Evershed RP, Dudd SN, Charters S, Mottram H, Stott AW, Raven A, et al Lipids as carriers of anthropogenic signals from prehistory. PhilTrans R Soc Lond B. 1999;354:19–31.

[30] Evershed RP. Organic residue analysis in archaeology: the archaeological biomarker revolution. Archaeometry. 2008;50(6):895–924.

[31] Evershed RP, Heron C, Goad LJ. Analysis of organic residues of archaeological origin by high-temperature gas chromatography and gas chromatography-mass spectrometry. Analyst. 1990;115(10):1339–42.

[32] Evershed RP, Vaughan SJ, Dudd SN, Soles JS. Fuel for thought? Beeswax in lamps and conical cups from Late Minoan Crete. Antiquity. 1997;71(274):979–85.

[33] Regert M, Colinart S, Degrand L, Decavallas O. Chemical alteration and use of beeswax through time: accelerated ageing test and analysis of archaeological samples from various environmental contexts. Archaeometry. 2001;43(4):549–69.

[34] Rageot M, Pêche-Quilichini K, Py V, Filippi JJ, Fernandez X, Regert M. Exploitation of Beehive Products, Plant Exudates and Tars in Corsica During the Early Iron Age. Archaeometry. 2016;58:315–32.

[35] Bernardini F, Tuniz C, Coppa A, Mancini L, Dreossi D, Eichert D, et al Beeswax as Dental Filling on a Neolithic Human Tooth. Plos one. 2012;7(9):e44904 10.1371/journal.pone.0044904 23028670 PMC3446997

[36] Ribechini E, Modugno F, Colombini MP, Evershed RP. Gas chromatographic and mass spectrometric investigations of organic residues from Roman glass unguentaria. J Chromatography A. 2008;1183(1):158–69. 10.1016/j.chroma.2007.12.090 18243222

[37] Rösch M, Sillman M, Ehrmann O, Liese-Kleiber H, Voigt R. Landnutzung im Umkreis der Zentralorte Asperg, Heuneburg und Ipf. Archäobotanische Untersuchungen und Modellberechnungen zum Ertragspotential des Ackerbaus. In: Krausse D, Beilharz D, editors. "Fürstensitze" und Zentralorte der frühen Kelten Abschlusskolloquium des DFG-Schwerpunktprogramms 1171 Forschungen und Berichte zur Vor- und Frühgeschichte in Baden-Württemberg. 2. Stuttgart: Konrad Theiss; 2009. p. 196–223.

[38] Berrio L, Wiethold J. Les macrorestes végétaux de Vix: synthèse des analyses carpologiques 1991–2014. In: Chaume B, editor. Nouvelles données sur le complexe aristocratique de Vix/le mont Lassois et son environnement (campagnes 2011–2017)forthcoming.

[39] Colonese AC, Hendy J, Lucquin A, Speller CF, Collins MJ, Carrer F, et al New criteria for the molecular identification of cereal grains associated with archaeological artefacts. Scientific Reports. 2017;7(1):6633 10.1038/s41598-017-06390-x 28747692 PMC5529501

[40] Krasutsky PA. Birch bark research and development. Nat Prod Rep. 2006;23(6):919–42. 629. 10.1039/b606816b 17119640

[41] Aveling EM, Heron C. Identification of Birch Bark Tar at the Mesolithic Site of Star Carr. Ancient Biomolecules. 1998;2:69–80.

[42] Rageot M, Théry-Parisot I, Beyries S, Lepère C, Carré A, Mazuy A, et al Birch Bark Tar Production: Experimental and Biomolecular Approaches to the Study of a Common and Widely Used Prehistoric Adhesive. Journal of Archaeological Method and Theory. 2019;26(1):276–312.

[43] McGovern PE, Luley BP, Rovira N, Mirzoian A, Callahan MP, Smith KE, et al Beginning of viniculture in France. Proceedings of the National Academy of Sciences. 2013;110(25):10147–52. 10.1073/pnas.1216126110 PMC3690885 23733937

[44] Guasch-Jané MR, Ibern-Gomez M, Andrés-Lacueva C, Jauregui O, Lamuela-Raventos RM. Liquid Chromatography with Mass Spectrometry in Tandem Mode Applied for the Identification of Wine Markers in Residues from Ancient Egyptian Vessels. Anal Chem. 2004;76:1672–7. 10.1021/ac035082z 15018566

[45] Körber-Grohne U. Pflanzliche Abdrücke in eisenzeitlicher Kerami—Spiegelbild damaliger Nutzplanzen? Fundberichte aus Baden-Württemberg. 1981;6:165–212.

[46] Valamoti SM, Mangafa M, Koukouli-Chrysanthaki C, Malamidou D. Grape-pressings from northern Greece: the earliest wine in the Aegean? Antiquity. 2007;81(311):54–61.

[47] Bouby L. L'agriculture dans le bassin du Rhône du Bronze final à l'Antiquité: Agrobiodiversité, économie, cultures. Toulouse: Archives d'écologie préhistorique; 2014.

[48] Correa-Ascencio M, Robertson IG, Cabrera-Cortés O, Cabrera-Castro R, Evershed RP. Pulque production from fermented agave sap as a dietary supplement in Prehispanic Mesoamerica. PNAS. 2014;11(39):14223–8. 10.1073/pnas.1408339111 PMC4191797 25225408

[49] Connan J. Use and trade of bitumen in antiquity and prehistory: molecular archaeology reveals secrets of past civilizations. Philos Trans R Soc Lond B Biol Sci. 1999;354 (1379):33–50.

[50] Saurel M. Le site protohistorique d’Acy-Romance (Ardennes). VI, Le temps et l’usage. Étude de céramique en pays rème (vers 400–30 av. J.-C.) et hypothèses autour du foyer, des boissons fermentées, de la chaux. Mémoire de la Société Archéologique Champenoise. 2017;22.

[51] Brun P, Chaume B. Les principautés celtiques du premier âge du Fer In: Garcia D, Guilaine J, editors. Pré et protohistoire de la France. Paris: Hermann; 2018 p. 74–388.

[52] Dietler M. Alkohol als verkörperte materielle Kultur. Vergleichende kulturanthropologische Überlegungen zum Konsum von Alkohol In: Stockhammer PW, Fries-Knoblach J, editors. Was tranken die frühen Kelten? BEFIM 1. Leiden: Sidestone Press; 2019 p. 299–319.

