# Supplementary material for: New insights into Early Celtic consumption practices: Organic residue analyses of local and imported pottery from Vix-Mont Lassois
Source: PLoS One. 2019 Jun 19;14(6):e0218001. doi: 10.1371/journal.pone.0218001 (PMC6583963; doi:10.1371/journal.pone.0218001)
Supplement: S1 Text — (DOCX) [file pone.0218001.s001.docx]

Vix-Mont Lassois is one of the rare Early Iron Age sites north of the Alps that has provided significant quantities of both local and imported Mediterranean high-quality fine ceramics over a considerable number of years of research. This provided the opportunity to carry out a large-scale investigation as part of the BEFIM project, whose objective was to study both kinds of wares in order to understand their specific meanings and functions within an Early Iron Age society.

One of the main aims of the BEFIM project was to clarify whether the imported pottery was used to imitate the Mediterranean (especially Greek) symposium, as has been supposed by scholars for many decades — meaning that the imported vessels would have been used in the same way as in their country of origin. The finds of transport amphorae and fine drinking and serving vessels in Early Iron Age sites north of the Alps were always thought to point to the importation and consumption of Mediterranean grape wine, as was the practice in Greek symposia. The selection of vessels forms for ORA was informed by this hypothesis, and sampling was primarily carried out on fine wares, especially forms potentially used for drinking, serving, mixing, and storage. In addition, we sampled and analysed a small number of coarse ware vessels to identify and compare their uses.

The ‘form’ and ‘function’ of many Greek vessels are known from ancient literary sources and images (1, 2, 3, 4). The potential function of pre- and protohistoric vessels has often been intuitively determined according to their overall shape (5). In this line of thinking, they have been compared to modern vessels and termed with corresponding names such as beakers, bottles, bowls etc. although their original terms and functions are unknown. Our vessel selection was guided by the necessity to carry out a comparative analysis between local and imported vessels of similar shape and/or assumed function:

**Drinking vessels**

a) local:

- beakers, bottle-shaped miniatures and goblet-shaped vessels (without imported equivalent)

- bowls with an everted rim as a possible equivalent to imported cups or kylikes.

b) imported:

- Greek cups or kylikes: according to ancient sources, these were used for drinking wine during the Greek-style symposium.

**Vessels used for storing, preparation and/or presentation**

a) local:

- bottle shaped large vessels were frequently found and could have been used for storing beverages or other liquids. In shape and perhaps in function they resemble the Greek amphorae. Regardless of their size, they are of rather fine fabrication and could therefore have had representative functions (as for Greek amphorae).

- large deep bowls or even coarse ware pots could have been used for mixing beverages, as local equivalents to the Greek krater.

b) imported:

- apart from their representative value, Greek fine ware amphorae were used to store liquids such as wine or olive oil.

- according to ancient literary sources, the function of kraters at Greek symposia was to mix wine with water and other ingredients.

**Vessels used for serving and ladling**

a) local:

- bottles (both hand-crafted and wheel-turned) and jugs (only wheel-turned) for serving drinks or other liquids. Both the painted hand-crafted specimens and the dark or brownish wheel-turned vessels were of high quality and representative function.

- small bowls, sometimes with an omphalos bottom, for ladling.

b) imported: none.

In total, 99 ceramic vessels of local and Mediterranean origin were selected for this study, including 83 locally made and 16 imported Mediterranean vessels. Fifty-one vessels (38 local and 13 imported) were taken from the archives of the old excavations, whereas 48 vessels (45 local and 3 imported) were recovered from modern excavations carried out since 2008.

**References S1**

1. Letzner W. Gebrannte Erde. Antike Keramik – Herstellung, Formen und Verwendung: Nünnerich-Asmus Verlag & Media, Mainz; 2015.
2. Heinemann A. Geschirr und Gesellschaft: Athenische Trinkgelage im 4. Jahrhundert v. Chr. In: Dickmann J-A, Heinemann A, editors. Vom Trinken und Bechern Das antike Gelage im Umbruch: Ausstellungskatalog Universität Freiburg; 2015. p. 18-22.
3. Grosser F. Flache Schalen - tiefe Becher: zwei Formen des Trinkens beim klassischen Weingelage. In: Dickmann J-A, Heinemann A, editors. Vom Trinken und Bechern Das antike Gelage im Umbruch: Ausstellungskatalog Universität Freiburg; 2015. p. 136-7.
4. Beilharz D, Krausse D. Symbole der Macht. Repräsentation in frühkeltischer Zeit. Welt der Kelten. Stuttgart: Jan Thorbecke Verlag; 2012.
5. Veit U. Zur Form und Funktion ur- und frühgeschichtlicher Gefäßkeramik: Eine semiotische Perspektive. Arch Inf. 1997;20(1):265-7.
