# Supplementary material for: New insights into Early Celtic consumption practices: Organic residue analyses of local and imported pottery from Vix-Mont Lassois
Source: PLoS One. 2019 Jun 19;14(6):e0218001. doi: 10.1371/journal.pone.0218001 (PMC6583963; doi:10.1371/journal.pone.0218001)
Supplement: S2 Text — (DOCX) [file pone.0218001.s002.docx]

The Early Iron Age settlement of Vix-Mont Lassois (Eastern France, Côte-d’Or, Burgundy) on the upper course of the River Seine held a key position for long-distance exchanges between the Mediterranean and the Atlantic Sea that was carried out via the Rivers Rhône, Saône and Seine. The hillfort settlement lies south of a geological elevation, which is cut through by the Seine. South of the Mont Lassois, the landscape opens into the wide space of a plateau. Living on top of the hill provided the advantages of a view of the open plain where the land trading routes passed by, and control of the point where the Seine, during the Iron Age, presumably became navigable. These benefits made Vix-Mont Lassois an attractive place ever since the Neolithic. In the Late Bronze Age there was first considerable settlement activity including a hillfort, ceramic depositions, and burials on the plain. About 300 years later, fortifications and burial places were partly re-used by the Late Hallstatt people.

The Early Iron Age settlement consisted of different settlement zones surrounded by a strong and complex system of fortifications (1). The ceramic vessels selected for organic residue analysis were recovered from the plateau settlement, from fortified settlement areas east (*Les Renards*) and west (*Champ Fossé*) of the Mont Lassois, and from a presumably open settlement in the plain south-east of the Mont Lassois (*Le Breuil*).

**The plateau contexts (Hallstatt D2-D3)**

The plateau settlement is situated on the upper plateau of the Mont Lassois (*plateau St-Marcel*, Fig 8, main manuscript). Geomagnetic measurements revealed a regular settlement pattern that was organised by pathways and enclosures and was possibly the result of intentional planning. The enclosures held buildings of different sizes and functions. Small rectangular dwellings and big granaries can be identified. On the eastern side of the plateau, there is an area differing from the rest of the location. According to the current stage of research, 5 large buildings with an apsidal ground plan dominated this area (1). The largest one was a hall-like construction, 35 m in length in its last building phase. It is outstanding for its architecture, proportions and division of space (2, 3, 4, 5). Most likely, it served representative functions. Recent excavations in the context of a collaborative research project (PCR Vix et son environnement, 2004-16) also confirm this, with finds of high quality ceramics of both local and Mediterranean origin. In view of the size of the building, one can postulate its function for assemblies and/or feasts. The presence of these outstanding finds and features supports the idea of the plateau settlement as an administrative centre and/or “residential area” of the Vix-Mont Lassois settlement complex. The pottery is of particular opulence, e.g. at the main building (apsidal house I), especially with regard to its tableware including 33% of local fine wheel-turned ceramics and 5% of imported vessels of Attic, Ionian and Massaliot origin (6).

A similar ceramic assemblage was discovered on the upper slope of the plateau during Lagorgette’s excavations in the 1930s (Fig 8, main manuscript). These complexes, located in close proximity to the “residential area” or so-called “palatial area”, have now been associated with the recently excavated area of the large apsidal buildings (7). However, the early date of the Lagorgette excavations results in a significant lack of information on the comprehensiveness of the preserved corpus of ceramic finds, especially since it comprises mostly complete or largely preserved vessels. Therefore, it must be assumed that, apart from the imported pottery, only a selection of local wares was kept.

Ceramics from both of these contexts consist of a broad spectrum of fine wares (imported, local wheel-turned, local handmade). Coarse wares from this area were not included in our study due to their uncharacteristic fragmentation. Indeed, their dating could not even be restricted to either the Bronze Age or Late Hallstatt period.

**Lower settlements near the ramparts (Hallstatt D2-D3)**

The recent excavation of the fortification on the lower slope of the plateau, conducted by the University of Zurich (PCR Vix, 2009-2016), revealed domestic/craft areas, with *Les Renards* and *Champ Fossé* as the most important contexts (Fig 8, main manuscript). Craft installations like ovens, several fireplaces, workshop spaces etc. and related features at *Les Renards* point to past activities of craftsmanship in these areas. Craft tools related to potential textile (e.g. a spindle) and ceramic production (firing misfires), as well as metallurgical activities (e.g. a preformed bronze fibula, bi-pyramidal iron ingots, etc.) were associated with *Les Renards* (8, 9) and also with some of the contexts at *Champ Fossé* (particularly the old excavations carried out by Joffroy) (10, 11, 12). Only local handmade ceramics, including both fine wares and coarse vessels, were studied from these areas (wheel-turned ceramics represent less than 1% NMI). These contexts include the highest concentration of well-dated coarse ware pottery from Vix-Mont Lassois (6).

**External area of Le Breuil (Hallstatt D3 / Hallstatt D3- La Tène A)**

The area of *Le Breuil* is situated on the plain, southeast of the Mont Lassois. It was excavated in 2013-15 (13) under the direction of B. Chaume (PCR 2014) and offers the first insights into a potentially much wider area of Early Iron Age settlement activities. The current state of excavation and evaluation of the evidence does not permit a sufficient understanding of the character of this part of the settlement and its association with the cemetery and sanctuary nearby. The discovery of wheel-turned ceramics is uncommon outside the plateau contexts and illustrates the presence of prestigious materials in the pits excavated, particularly in *Feature 28*. So far, there are no clear architectonic structures and imported pottery from *Le Breuil*. The presence of wheel-turned ceramics might be explained by i) their production at/or in the vicinity of Vix, ii) their later chronological appearance (involving wider diffusion?) and/or iii) their proximity to the necropolis.

Our analyses from *Le Breuil* included 13 local vessels, most of which are fine ceramics (handmade and wheel-turned), and only one coarse ware vessel due to the low numbers excavated.

**References S2**

1. Chaume B. Le complexe aristocratique de Vix/le mont Lassois. Bull Arch et Hist Châtillonnais 2013;5(31-45).
2. Chaume B, Nieszery R, Reinhard W. La partie médiane et la façade à antes du grand bâtiment absidial. In: Chaume B, Mordant C, editors. Le complexe aristocratique de Vix : nouvelles recherches sur l'habitat, le système de fortification et l'environnement du mont Lassois. Dijon: Éditions universitaires; 2011. p. 430-78.
3. Chaume B, Nieszery R, Reinhard W. Der Mont Lassois – ein frühkeltischer Fürstensitz im Burgund. Die Welt der Kelten - Zentren der Macht - Kostbarkeiten der Kunst. Stuttgart: Jan Thorbecke Verlag; 2012. p. 132-8.
4. Chaume B, Nieszery R, Reinhard W. L’enclos aux grands bâtiments absidiaux (campagnes de fouilles de 2008-2012). In: Chaume B, editor. Nouvelles données sur le complexe aristocratique de Vix/le mont Lassois et son environnement (campagnes 2011-2017) forthcoming a.
5. Buchsenschutz O, Mötsch A, editors. Réflexions sur l’architecture monumentale à la fin du premier âge du Fer. In: Villard-Le Tiec A, Menez Y, Maguer P, editors. Architecture de l’âge du Fer en Europe occidentale et centrale Actes du 40e colloque international de l'Association Française pour l’Étude de l’Âge du Fer AFEAF; 2018. p. 259-271.
6. Bardel D. Société, économie et territoires à l’âge du Fer dans le Centre-Est de la France. Analyse des corpus céramiques des habitats du Hallstatt D – La Tène A (VIIe - Ve siècle av. J.-C.): PhD thesis, Université de Bourgogne; 2012.
7. Chaume B, Mordant C. Le complexe aristocratique de Vix. In: Chaume B, Mordant C, editors. Nouvelles recherches sur l’habitat et le système de fortifications. Dijon: Editions universitaires; 2011. p. 867.
8. Winkler A, Della Casa P. Une zone artisanale hallstattienne sur le site princier de Vix (Côte-d’Or) au lieu-dit Les Renards. Bilan intermédiaire. In: Marion S, Deffressigne S, Kaurin J, Bataille G, editors. Production et proto-industrialisation aux Âges du Fer Perspectives sociales et environnementales Actes du 39e colloque international de l’AFEAF; 4-17 mai 2015; Nancy. Bordeaux: Ausonius éditions; 2017. p. 693-700.
9. Winkler A. Status des Handwerks und des Handwerkers in der Späthallstattzeit. Eine Überlegung am Fallbeispiel der Grabung eines Handwerksareals am Mont Lassois (Burgund, Frankreich). In: Karl R, Leskovar J, editors. Interpretierte Eisenzeiten Fallstudien, Methoden, Theorie Tagungsbeiträge der 7 Linzer Gespräche zur interpretierten Eisenzeitarchäologie Studien zur Kulturgeschichte von Oberösterreich 47: Linz; 2017. p. 151-62.
10. Chaume B. Vix et son territoire à l’âge du Fer. Fouilles du mont Lassois et environnement du site princier. Montagnac: Éditions Mergoil; 2001.
11. Schäppi K, Ballmer A, Della Casa P. Campagne triennale 2009-2011 de l’Université de Zurich, Abt. Ur- und Frühgeschichte, au mont Lassois. Rapport d’activité du PCR Vix et son Environnement 2009-2011. Dijon: Université de Bourgogne; 2011.
12. Ballmer A, Schäppi K, Della Casa P. Les fouilles du Champ Fossé à Vix (Côte-d’Or). Campagnes 2009-2014 forthcoming.
13. Chaume B, Nieszery R, Reinhard W. Les fouilles du site du Breuil. In: Chaume B, editor. Nouvelles données sur le complexe aristocratique de Vix/le mont Lassois et son environnement (campagnes 2011-2017) forthcoming b.
