# Supplementary material for: New insights into Early Celtic consumption practices: Organic residue analyses of local and imported pottery from Vix-Mont Lassois
Source: PLoS One. 2019 Jun 19;14(6):e0218001. doi: 10.1371/journal.pone.0218001 (PMC6583963; doi:10.1371/journal.pone.0218001)
Supplement: S3 Text — (DOCX) [file pone.0218001.s003.docx]

**Control samples and exogenous contamination**

Samples taken from the external surfaces of selected vessels and soils from the burial contexts served as controls for exogenous contamination. Soil samples were taken from the *Les Renards* and *Champ Fossé* contexts, where excavations are still ongoing, hence providing the opportunity to obtain sediment. No trace of hopanoids were identified in the control samples. Less than 12 µg of lipid per g of sediment were recovered. Long chain even-numbered fatty acids (C_26:0_ and C_28:0_), *n-*alcohol (C26 dominant), esters (hexacosanyl derivates) and *n-*alkanes were the main compounds identified. Trace amounts of miliacin were identified in the soil samples taken from *Les Renards,* which were taken into consideration when miliacin was present in vessels from this context (Fig S3-1).


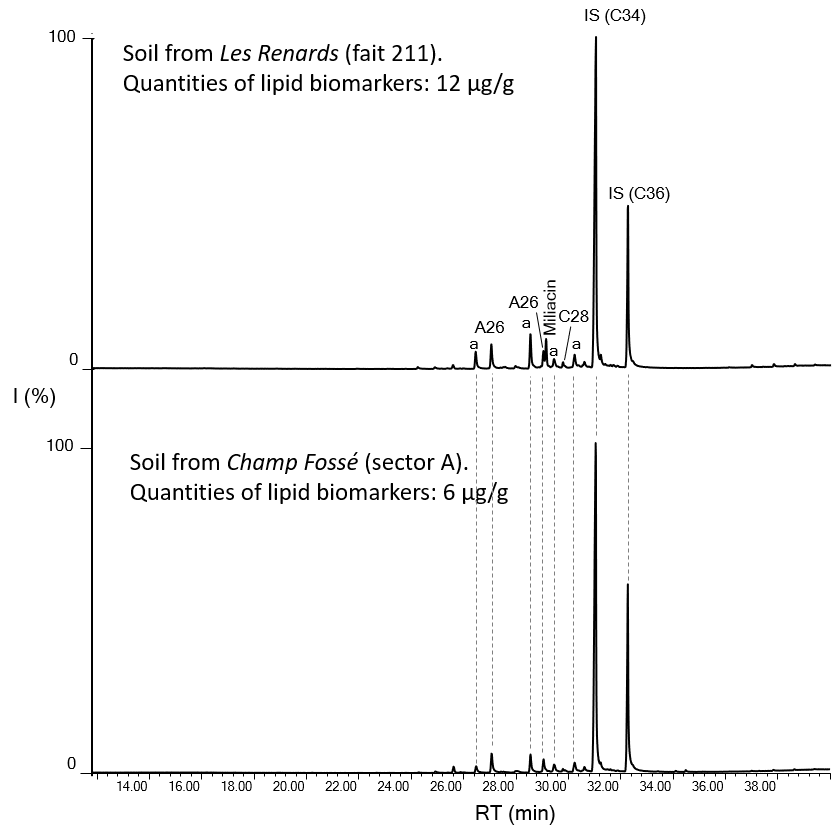


Fig S3-1: Soil control samples from the *Les Renards* and *Champ Fossé* contexts. Cx=fatty acids with x number of carbon atoms; Ax=n-alcohols with x number of carbon atoms; a=n-alkanes

Unfortunately, sediment samples from the plateau and the external area of *Le Breuil* were not available. Samples taken from the exterior surface of selected vessels served as controls. These showed traces of lipids, particularly phthalates, palmitic and stearic acids, which can be attributed to post-excavation handling. Cholesterol was also identified in the control sample from the plateau (VIX-ALT-012, Fig S3-2). This compound, a marker for the presence of animal fat, was also identified in all of the Attic ceramics from the plateau context but never in association with other animal fat markers, such as specific triglycerides or high quantities of stearic acid. In view of this, cholesterol in these vessels is likely to be a contaminant.


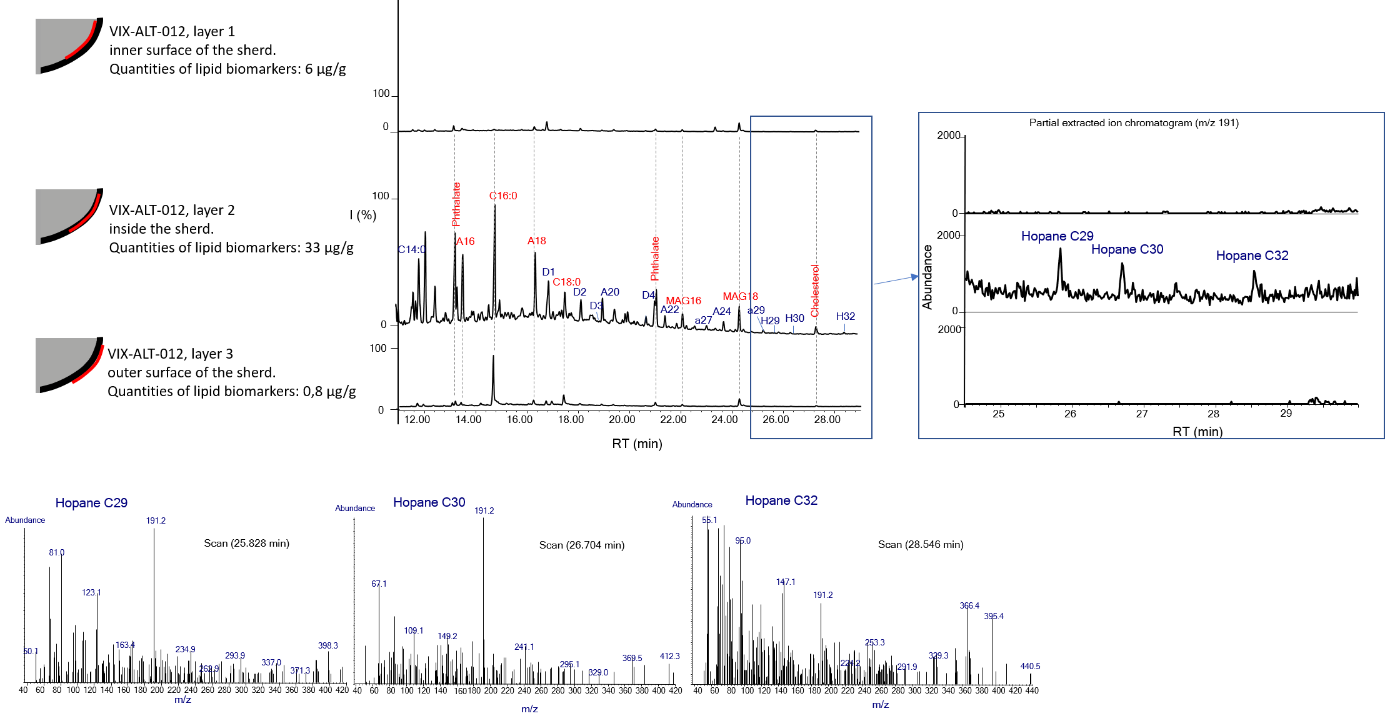


Fig S3-2: Control sample from the plateau context (VIX-ALT-012). Red: possible exogenous contamination. Blue: archaeological content. D1=Hydrocarbon diterpene (phenanthrene); D2=methyl dehydroabietate; D3=dehydroabietic acid; D4=7-oxo-dehydroabietic acid; Hx=hopanoids with x number of carbon atoms; ; Cx:y=fatty acids with x number of carbon atoms and y denoting unsaturations; Ax=n-alcohols with x number of carbon atoms; ax=n-alkanes with x number of carbon atoms.

**Description of the biomarkers present in the vessels from Vix-Mont Lassois**

**Wax esters**

Long chain palmitic esters with an even number of carbon atoms ranging from C40 up to C50 were identified in a large number of samples (n=40) (S4 Tables 1, 3-5). The latter, together with their hydrolysis products (palmitic acid and even-numbered *n*-alcohols comprising 22 to 34 carbon atoms), a series of odd-number *n*-alkanes (C_27_ major) and often with saturated long chain even-numbered fatty acids (C_22:0_ to C_28:0_, maximising at C_24:0_) are characteristic of beeswax (1, 2). The presence of other suites of long chain palmitic esters (C32-C42) but also stearic and arachidic esters, suggest the presence of plant waxes (3; S4 Tables 1-5) in 35 samples.

**Fatty acids and triglycerides: animal origin**

A distribution of saturated triglycerides (TAGs) containing up to 54 carbon atoms was identified in 8 samples and shows the presence of animal fats (4, 5). Based solely on molecular markers, it was difficult to identify the origin of the animal fat when samples contained a narrow distribution of TAG signatures. Indeed, hydrolytic processes from natural and anthropogenic degradation preferentially affect the shorter TAGs, thus preventing secure distinction between an adipose and dairy origin (6). Dairy products could be identified when a wide TAG distribution, comprising TAGs with 36 to 54 carbon atoms, was present. Identifications were confirmed by measuring the isotopic values (δ^13^C) of the palmitic and stearic fatty acids (4, 7). To avoid erroneous interpretations due to mixtures with other substances such as beeswax and plant by-products, isotopic analyses were carried out only when C_16:0_ and C_18:0_ were the major fatty acids in the mixture (11 samples, see S4 Tables 1, 3- 5).

**Fatty acids and triglycerides: plant origin**

Significant quantities of oleic acid were identified in 22 samples. This molecular marker, together with other C18 unsaturated compounds and palmitic acid are the major fatty acids in plant oils. A higher abundance of oleic acid over stearic acid could occur in some modern animal adipose tissues, but it is extremely rare to have such high oleic to stearic acid ratios in archaeological samples. Unsaturated fatty acids are more susceptible to degradation processes than saturated fatty acids, primarily through oxidation (8, 9). The ratio of palmitic to stearic acid in the samples thought to have a plant origin is higher than 2:1 (higher than 4:1 for 10 of them). This ratio could be indicative of a plant oil (10). However, this argument is less persuasive for archaeological residues due to the differential degradation processes of fatty acids. Indeed, longer chain fatty acids degrade more rapidly in most burial contexts (9).

Residues were categorised as oily plants if the quantity of oleic acid was above or equal to stearic acid.

The presence of linoleic acid, identified in 12 samples, confirms this origin. Plant oils can only be detected if they occur in high concentrations, and preservation conditions must be favourable as they are more subject to degradation than animal fats (8). For these reasons, the chemical signatures identified are more consistent with oily plants, although oleic acid and linoleic acid are present in modern cereals and vegetables. Furthermore, unsaturated TAGs namely 54:3, 52:2 and 50:1, were identified in 3 of the samples. This suite of TAGs is primarily present in plant oils. The presence of unsaturated triacylglycerols is extremely rare in archaeological residues, as they are rarely preserved. Only plants that contain high quantities of oils, of which unsaturated TAGs are a primary constituent, will lead to preservation and consequently identification. Analysis of reference plant chemical signatures using the same analytical conditions as for the archaeological samples showed that the molecular compounds’ presence and their respective quantities are mostly consistent with those found in Mediterranean olive oil. Hence, this is the most likely source of the plant oil identified (Table S3-1).

Table A: Analytical results of modern plant oils extracted using the same protocol as for archaeological samples. Plant oil references were allowed to stand in ceramics for one week. The choice of the local plant oils was based on the archaeobotanical record.

**Di- and triterpenes**

Diterpenoids were detected in 11 samples (S4 Tables 1-2), particularly dehydroabiectic acid, 7-oxodehydroabietic, abieta-6,8,11,13-tetraen-18-oic acid, and retenes. These markers, formed by direct or indirect degradation of abietic acid, are characteristic of resin or tar obtained from *Pinaceae* (11, 12). In addition, α- and β-seco-dehydroabietic acids, pimaric acid and isopimaric acid were identified in significant amounts in some of these vessels. Their presence is characteristic of a *Pinus* origin (11).

Miliacin, a triterpenoid marker of *Panicum miliaceum* (common millet), was identified in 18 samples and indicates that this cereal was consumed here (13, 14).

The presence of betulin and lupeol, the major triterpenoid biomarkers of birch bark (15, 16), and occasionally betulone, which results from the oxidation of betulin over the time (15), were identified in 4 samples. They suggest the presence of products made from birch bark or birch sap. In 3 other samples, additional biomarkers (erythrodiol and betulinic acid) and degradation markers were identified, including allobetulin, allobetul-2-ene and α-betulin I. These are characteristic of thermal degradation during specific tar production (17).

**Bacteriohopanes: bacteria fermented alcoholic beverage?**

A series of compounds characterised by a base peak at m/z 191 and molecular ions (M+.) 398 (C_29_H_50_) and 412 (C_30_H_52_), and occasionally 426 (C_31_H_54_), 440 (C_32_H_56_), 454 (C_33_H_58_) and 468 (C_34_H_60_), corresponding to hopanes, were identified in 39 vessels. Bacteriohopanes are ubiquitous compounds in the environment and are regularly observed in wet soils, mostly in peat environments. They can result from bacterial activity. In the study of Vix, we confidently excluded exogenous contamination by analysing appropriate control samples. (See control, Fig S3-1 and Fig S3-2). These included samples taken from the external surface of the vessels and soil samples taken from the burial contexts. Bacteriohopanoids were absent in all of the control samples tested. Bacteriohopanoids were present only in the powdered ceramic samples taken from the interior surface of the vessels. Furthermore, the wetter soil at Vix, where bacteriohopanoids are more likely to be present in the sediment, is found close to the Seine river (Fig 8, main manuscript) and corresponds to the contexts of *“Les Renards”* and “*Le Breuil”* where hopanoids are absent in the vessels tested. This additionally supports our finding that hopanoids identified in the vessels did not originate from the soil.

Depending on the archaeological context and the artefact in which they are found, hopanoids can point to the presence of different substances such as bitumen (18) or fermented alcoholic beverages (19). Fermentation bacteria of type *Zymomonas mobilis* develop a membrane rich in hopanoids to resist ethanol stress and low pH (19). Hopanoid biomarkers of this bacterium could occur as a result of the fermentation of sugar from plant by-products such as palm, beer and a wide range of other plant sap and fruit derived beverages (19, 20).

Our interpretation, associating hopanoids with fermented beverages rather than bitumen or plankton is based on the archaeological context and the specific techno-typology of the vessels in which these markers were found (namely fine ceramics which have been attributed drinking and serving functions). This interpretation is further supported by experimental work using use-wear analysis, which showed that pitting could result from fermentation processes (21). Similar pitting was identified on the interior surface of bottles from Vix-Mont Lassois, in which bacteriohopanoids were also present.

**Short-chain carboxylic compounds: fruit products?**

Following KOH or BF_3_ extraction, short-chain carboxylic compounds were identified in 20 samples. These included succinic, fumaric, malic and tartaric acids. Tartaric acid, identified in 16 samples, is usually considered to be a grape product/wine marker because of its high concentration in grapes (22, 23, 24, 25, 26) in contrast to other fruits available in Europe during EIA (Table S3-2). Indeed, a high concentration of tartaric acid is also known in some tropical plants (26) or in Chinese hawthorn tree fruit (27). When present in the Vix vessels, tartaric acid was mostly identified together with other short-chain carboxylic compounds, including succinic acid (S4 Tables 2- 5), which are known wine fermentation markers (22, 23). In 4 of the vessels containing tartaric acid, the only other compound present was malic acid. Malic, succinic and fumaric acids were also identified in the absence of tartaric acid in some vessels, suggesting 1) the presence of fruit products other than grape, or 2) that grape wine had been present but tartaric acid was lost due to advanced degradation. When this was the case, an interpretation of grape wine was not assigned.

Table B: Analytical results of modern fruit products extracted using the same protocol as archaeological samples. Fruit product references were stored in ceramic for one week prior to analysis.

**References S3**

1. Evershed RP, Vaughan SJ, Dudd SN, Soles JS. Fuel for thought? Beeswax in lamps and conical cups from Late Minoan Crete. Antiquity. 1997;71(274):979-85.
2. Regert M, Colinart S, Degrand L, Decavallas O. Chemical alteration and use of beeswax through time : accelerated ageing test and analysis of archaeological samples from various environmental contexts. Archaeometry. 2001;43(4):549-69.
3. Ribechini E, Modugno F, Colombini MP, Evershed RP. Gas chromatographic and mass spectrometric investigations of organic residues from Roman glass unguentaria. J Chromatography A. 2008;1183(1):158-69.
4. Regert M. Analytical strategies for discriminating archeological fatty substances from animal origin. Mass Spectrometry Reviews. 2011;30(2):177-220.
5. Evershed RP, Heron C, Goad LJ. Analysis of organic residues of archaeological origin by high-temperature gas chromatography and gas chromatography-mass spectrometry. Analyst. 1990;115(10):1339-42.
6. Evershed RP, Dudd SN, Copley MS, Berstan R, Stott AW, Mottram H, et al. Chemistry of archaeological animal fats. Accounts Of Chemical Research. 2002;35(8).
7. Evershed RP, Dudd SN, Copley MS, Mutherjee A. Identification of animal fats via compound specific δ13C values of individual fatty acids: assessmentsof results for reference fats and lipid extracts of archaeological pottery vessels. Documenta Praehistorica. 2002;XXIX:73-96.
8. Regert M, Bland HA, Dudd SN, Bergen PFV, Evershed RP. Free and bound fatty acid oxidation products in archaeological ceramic vessels. Proc R Soc Lond B. 1998;265:2027-32.
9. Evershed RP, Dudd SN, Charters S, Mottram H, Stott AW, Raven A, et al. Lipids as carriers of anthropogenic signals from prehistory. PhilTrans R Soc Lond B. 1999;354:19-31.
10. Romanus K, Baeten J, Poblome J, Accardo S, Degryse P, Jacobs P, et al. Wine and olive oil permeation in pitched and non-pitched ceramics: relation with results from archaeological amphorae from Sagalassos, Turkey. Journal of Archaeological Science. 2009;36:900-9.
11. Helwig K, Monahan V, Poulin J. The Identification of Hafting Adhesive on a Slotted Antler Point from a Southwest Yukon Ice Patch. American Antiquity. 2008;73(2):279-88.
12. Steigenberger G, Herm C. Natural resins and balsams from an eighteenth-century pharmaceutical collection analysed by gas chromatography/mass spectrometry. Analytical and Bioanalytical Chemistry. 2011;401(6):1771-84.
13. Jacob J, Disnar J-R, Arnaud F, Chapron E, Debret M, Lallier-Vergès E, et al. Millet cultivation history in the French Alps as evidenced by a sedimentary molecule. Journal of Archaeological Science. 2008;35(3):814-20.
14. Heron C, Shoda S, Breu Barcons A, Czebreszuk J, Eley Y, Gorton M, et al. First molecular and isotopic evidence of millet processing in prehistoric pottery vessels. Scientific Reports. 2016;6:38767.
15. Aveling EM, Heron C. Identification of Birch Bark Tar at the Mesolithic Site of Star Carr. Ancient Biomolecules. 1998;2:69-80.
16. Krasutsky PA. Birch bark research and development. Nat Prod Rep. 2006;23.
17. Rageot M, Théry-Parisot I, Beyries S, Lepère C, Carré A, Mazuy A, et al. Birch Bark Tar Production: Experimental and Biomolecular Approaches to the Study of a Common and Widely Used Prehistoric Adhesive. J Archaeol Method Theory. 2018 https://doi.org/10.1007/s10816-018-9372-4.
18. Connan J. Use and trade of bitumen in antiquity and prehistory: molecular archaeology reveals secrets of past civilizations. Philos Trans R Soc Lond B Biol Sci. 1999;354 (1379):33–50.
19. Correa-Ascencio M, Robertson IG, Cabrera-Cortés O, Cabrera-Castro R, Evershed RP. Pulque production from fermented agave sap as a dietary supplement in Prehispanic Mesoamerica. Proc Natl Acad Sci USA. 2014;111(39):14223-8.
20. Roffet-Salque M, Dunne J, Altoft DT, Casanova E, Cramp LJE, Smyth J, et al. From the inside out: Upscaling organic residue analyses of archaeological ceramics. Journal of Archaeological Science. 2017;16: 627-40.
21. Saurel M. Le site protohistorique d’Acy-Romance (Ardennes). VI, Le temps et l’usage. Étude de céramique en pays rème (vers 400-30 av. J.-C.) et hypothèses autour du foyer, des boissons fermentées, de la chaux. Mémoire de la Société Archéologique Champenoise. 2017;22.
22. Pecci A, Giorgi G, Salvini L, Cau Ontiveros MÁ. Identifying wine markers in ceramics and plasters using gas chromatography–mass spectrometry. Experimental and archaeological materials. Journal of Archaeological Science. 2013;40(1):109-15.
23. Garnier N, Valamoti SM. Prehistoric wine-making at Dikili Tash (Northern Greece): Integrating residue analysis and archaeobotany. Journal of Archaeological Science. 2016;74:195-206.
24. Guasch-Jané MR, Ibern-Gomez M, Andrés-Lacueva C, Jauregui O, Lamuela-Raventos RM. Liquid Chromatography with Mass Spectrometry in Tandem Mode Applied for the Identification of Wine Markers in Residues from Ancient Egyptian Vessels. Anal Chem. 2004;76:1672-7.
25. McGovern PE, Luley BP, Rovira N, Mirzoian A, Callahan MP, Smith KE, et al. Beginning of viniculture in France. Proceedings of the National Academy of Sciences. 2013;110(25):10147-52.
26. Barnard H, Dooley AN, Areshian G, Gasparyan B, Faull KF. Chemical evidence for wine production around 4000 BCE in the Late Chalcolithic Near Eastern highlands. Journal of Archaeological Science. 2011;38(5):977-84.
27. McGovern PE, Zhang J, Tang J, Zhang Z, Hall GR, Moreau RA, et al. Fermented beverages of pre- and proto-historic China. Proceedings of the National Academy of Sciences of the United States of America. 2004;101(51):17593-8.
